# Effects of a compound from the group of substituted thiadiazines with hypothermia inducing properties on brain metabolism in rats, a study *in vivo* and *in vitro*

**DOI:** 10.1371/journal.pone.0180739

**Published:** 2017-07-05

**Authors:** O. B. Shevelev, N. B. Illarionova, D. V. Petrovski, A. P. Sarapultsev, O. N. Chupakhin, M. P. Moshkin

**Affiliations:** 1 The federal research center Institute of Cytology and Genetics Siberian Branch of the Russian Academy of Sciences, Novosibirsk, Russia; 2 Institute of Immunology and Physiology of the Ural Branch of the Russian Academy of Sciences, Ekaterinburg, Russia; 3 Federal State Autonomous Educational Institution of Higher Professional Education, Ural Federal University named after the first President of Russia B. N. Yeltsin, Ekaterinburg, Russia; 4 Institute of Organic Synthesis, Ural Division of Russian Academy of Sciences, Ekaterinburg, Russia; 5 Tomsk State University, Department of Vertebrate Zoology, Tomsk, Russia; National University of Singapore, SINGAPORE

## Abstract

The aim of the present study was to examine how administration of a compound of 1,3,4- thiadiazine class 2-morpholino-5-phenyl-6H-1,3,4-thiadiazine, hydrobromide (L-17) with hypothermia inducing properties affects the brain metabolism. The mechanism by which L-17 induces hypothermia is unknown; it may involve hypothalamic central thermoregulation as well as act via inhibition of energy metabolism. We tested the hypothesis that L-17 may induce hypothermia by directly inhibiting energy metabolism. The study *in vivo* was carried out on Sprague-Dawley adult rats. Two doses of L-17 were administered (190 mg/kg and 760 mg/kg). Brain metabolites were analyzed in control and treated groups using magnetic resonance spectroscopy, along with blood flow rate measurements in carotid arteries and body temperature measurements. Further *in vitro* studies on primary cultures from rat hippocampus were carried out to perform a mitochondria function test of L-17 pre-incubation (100 μM, 30 min). Analysis of brain metabolites showed no significant changes in 190 mg/kg treated group along with a significant reduction in body temperature by 1.5°C. However, administration of L-17 in higher dose 760 mg/kg provoked changes in brain metabolites indicative of neurotoxicity as well as reduction in carotid arteries flow rate. In addition, a balance change of excitatory and inhibitory neurotransmitters was observed. The L-17 pre-incubation with cell primary cultures from rat brain showed no significant changes in mitochondrial function. The results obtained in the study indicate that acute administration of L-17 190 mg/kg in rats induces mild hypothermia with no adverse effects onto brain metabolism.

## Introduction

Therapeutic hypothermia is an intentional lowering of core body temperature that is used in the clinic. The recovery benefit of mild (33–36°C), to moderate (28–33°C) therapeutic hypothermia is reported for neurological complications associated with cardiac arrest [1] and hypoxic ischemic neonatal encephalopathy [2] as well as in animal studies for traumatic brain injury and stroke [3–5]. The mechanisms of therapeutic hypothermia action may be attributed to the preservation of metabolic stores and lowering of excitatory neurotransmitters and other cytotoxic products release as well as anti-inflammatory processes [6, 7]. Therapeutic hypothermia may be achieved by external methods of cooling by using cooling systems, like cooling blankets, helmets, cold water or ice. However human body temperature protective mechanisms, like shivering thermogenesis, increases in heart rate and metabolism and redirection of blood flow from limbs and skin to vital organs, compromise these methods of achieving hypothermia results. In addition, an unintentional overcooling is a possible outcome.

Body heat in endotherms is produced mostly by metabolic processes. Metabolic failure unavoidably leads to suppression in thermogenesis. Thermoregulation occurs via brain-driven mechanisms that involve peripheral and central temperature sensory system, thermoreceptors and central system—primarily hypothalamus for maintaining stable core body temperature. Some chemicals are also known to reduce body temperature by central neural pathways via cannabinoid receptors [8], opioid receptors [9], dopamine receptors [10], serotonin receptors [11], neurotensin receptors [12] and by local tissue and cell action for example with adenosine, 2-DG [13, 14].

Derivatives of 1,3,4-thiadiazine class compounds constitute an important class of heterocycles, that have attracted much synthetic interest due to their wide range of biological activities, such as antibacterial and antifungal [15], cardiovascular and antiaggregant [16, 17], antiviral [18, 19]. Moreover, *in vivo* studies have shown anti-inflammatory activity of 1,3,4-thiadiazine derivatives (2-morpholino-5-phenyl-6H-1,3,4-thiadiazine hydrobromide, compound L-17) in experimental acute pancreatitis [20] and experimental cardiac infarction [21–22]. One of the most interesting effects of L-17 intraperitoneal injection is to reduce mouse body temperature (unpublished data, Boiko ER et. al. and our data S1 Fig, reduction by 8°C in 1 h 140 mg/kg L-17, S1 Table). The cause of this hypothermic reaction may be either by direct inhibition of cellular metabolism or changes in the body thermoregulatory set point via central regulation. To test the hypothesis of L-17 body temperature reduction by means of a direct effect onto the energy metabolism in experiments on Sprague-Dawley rats we examined effects of L-17 treatment on most abundant brain metabolites *in vivo* and mitochondrial function *in vitro*, changes in body temperature and blood flow velocity in carotid arteries. The use of a lower L-17 dose (190 mg/kg) led to a decrease in body temperature, but did not associate with significant changes in brain metabolism or blood flow in carotids. The use of a higher dose of L-17 (760 mg/kg) led to a reduction of blood flow in carotid arteries and changes in brain metabolites pattern. Studies *in vitro* indicated no effect of L-17 on mitochondrial function. Taken together, the data shows L-17 induced reduction in the rat body temperature possibly involving regulation via central thermoregulation system.

## Materials and methods

### Chemicals

The molecule 2-morpholino-5-phenyl-6H-1,3,4-thiadiazine hydrobromide (compound L-17) from the family of 1,3,4-thiadiazine compounds was synthesized at Ural Federal State University in Russia by cyclocondensation of α-bromoacetophenone with the original morpholine-4-carbothionic acid hydrazide molecule [23]. L-17 formula is C_13_H_16_BrN_3_OS, it contains 1,3,4-thiadiazine ring, morpholine and benzen ring. L-17 dissolves in water 20–40 mg/ml RT, has a pH = 3.3.

Oligomycin, FCCP, and a mix of rotenone and antimycin A were purchased from Seahorse XF Cell Mito Stress Test Kit (Seahorse Bioscience).

### Experimental animals and husbandry conditions

All animals were handled according to the recommendations in the Guide for the Care and Use of Laboratory Animals of the Russian National Center of Genetic Resources of Laboratory Animals on the basis of specific pathogen-free Vivarium (SPF). The experimental protocol was approved by the Bioethical Committee of the The federal research center Institute of Cytology and Genetics Siberian Branch of the Russian Academy of Sciences, Novosibirsk, Russia (permit No 27, April 24, 2015 and No 22.1, May 30, 2014). The study was conducted at the Center for Genetic Resources of Laboratory Animals at the Institute of Cytology and Genetics, Siberian Branch, Russian Academy of Sciences (RFMEFI61914X0005 and RFMEFI62114X0010). For the spectroscopy studies 18 Sprague-Dawley male rats aged from 12 to 13 weeks were used. Body temperature in rats and mice was measured with implantable temperature data loggers (model TDL3-28, EMBI RESEARCH LLC, Novosibirsk, Russia, 0.8 g, 5 min recording interval, accuracy 0.1°C), which were implanted into the abdomen under isoflurane anesthesia a week before and were fixed for the whole duration of the experiment (16 Sprague-Dawley rats and 6 BALB/c mice). Animals were housed in individually ventilated cages, with one animal per cage. The cages had a height of 20.5 cm and an area of 929 cm^2^ (OptiRAT cage; Charles River Laboratories). Water and Chara SPF granulated forage for laboratory rodents (Assortiment-Agro, Puschino, Russia) were given ad libitum. Rodents were kept in artificial day-night regime (14-hour light/10-hour darkness), at a temperature of 22 to 24°C, and humidity of 40 to 50%. Dry dedusted wood shavings (Albion, Novosibirsk, Russia) were used as litter. The food and litter were autoclaved before use. Drinking water was deionized with a Millipore NF-C8674 (Merck Millipore) and supplemented with Severyanka mineral additive (Eko-proect, St. Petersburg, Russia).

### Magnetic resonance imaging (MRI)

All medications were intraperitoneally injected 2 h prior to magnetic resonance imaging studies.

1. Control group (physiologic saline) (N = 7).
2. Low dose L17 (190 mg/kg) (N = 7).
3. High dose L17 (760 mg/kg) (N = 4).

Measurements of blood parameters and neurometabolites were carried out on a horizontal tomograph with a magnetic field of 11.7 Tesla (Bruker, Biospec 117/16 USR, Germany). Diameter and volumetric flow rate of carotid arteries, and metabolites levels of rat’s cerebral cortex were measured using ^1^H radio frequency coils. Rats were immobilized with a gas anesthesia (Isofluran; Baxter Healthcare Corp., Deerfield, IL) using a Univentor 400 Anesthesia Unit (Univentor, Zejtun, Malta). The animal body temperature was maintained with a water circuit installed into the table bed of the tomograph, which was maintained at 30°C on its surface. A pneumatic respiration sensor (SA Instruments, Stony Brook, NY) was placed under the lower body part, which allowed for the control of anesthesia depth.

### Magnetic Resonance Angiography (MRA)

Images and linear blood flow velocity in rat main blood vessels were recorded with transmitter volume (500.3 MHz; distribution, 72/89 mm) and receiver surface (500.3 MHz; 123×64×31 mm) ^1^H radiofrequency coils. To determine the diameter of blood vessels (carotid arteries) GEFC (Gradient echo flow compensated) method was used with parameters of a pulse sequence TE = 3.2 ms, TR = 15 ms (three-dimensional vessels image FOV 4×4×4 cm and a matrix size of 256×256×128 dots). The blood flow rate was determined using FLOWMAP method (flow-compensated gradient echo method) with the pulse sequence parameters TE = 6 ms, TR = 20 ms (1 mm thick slices with a field of view of 4×4 cm and a matrix size 256×256 dots).

### In vivo magnetic resonance spectroscopy (^1^H-MRS)

**Fig 1:**
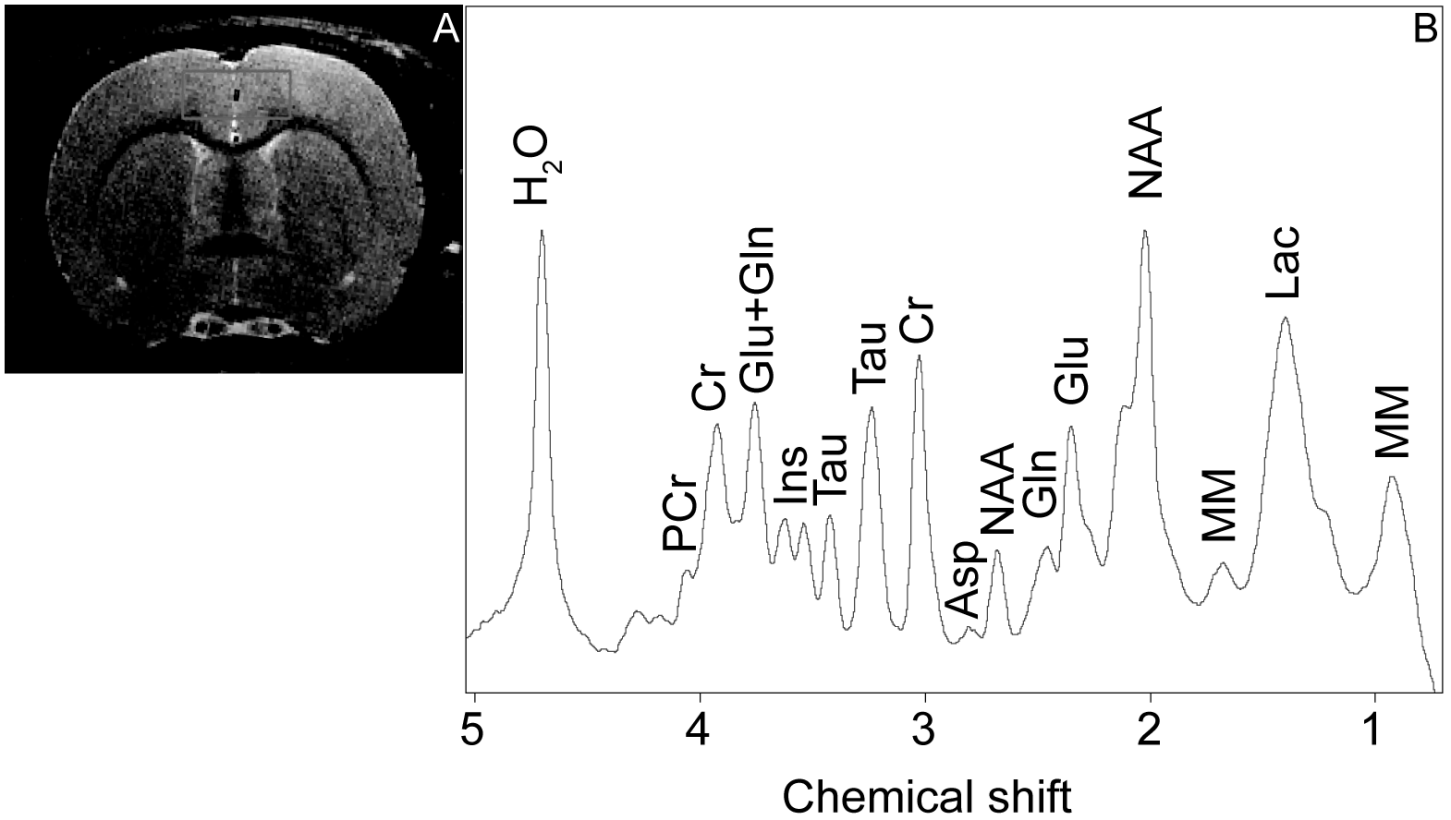
A Position of a voxel during ^1^H NMR spectroscopy of the brain cortex, and B a representative ^1^H NMR spectrum. Ins, myo-inositol; PCr, phosphocreatine; Cr, creatine; Glu, glutamic acid; Gln, glutamine; Tau, taurine; Asp, aspartate; NAA, N-acetylaspartate; GABA, gamma-aminobutyric acid; MM, macromolecules; Ala, alanine; and Lac, lactate.

All proton spectra of the rat brain cortex were recorded with transmitter volume (500.3 MHz; distribution, 72/89 mm) and receiver surface (500.3 MHz; 123×64×31 mm) ^1^H radiofrequency coils. High-resolution T_2_-weighted images of the rat brain (section thickness, 0.5 mm; field of view, 2.5×2.5 mm; matrix, 256×256 dots) were recorded by RARE (rapid with relaxation enhancement) with the pulse sequence parameters TE = 11 ms and TR = 2.5 seconds for the correct positioning of the spectroscopic voxels (1.6×4.0×3.0 mm). Fig 1A shows the position of the voxel in an axial section. All proton spectra were recorded by spatially localized single-voxel STEAM (stimulated echo acquisition mode) spectroscopy with the pulse sequence parameters TE = 3 ms and TR = 5 seconds and 100 accumulations. Uniformity of the magnetic field was tuned within the selected voxel using FastMap (Bruker) before each spectroscopic recording. The water signal was inhibited with a variable pulse power and optimized relaxation delays (VAPOR) sequence.

### Processing of ^1^H spectra

The experimental ^1^H magnetic resonance spectra were processed and the quantitative composition of metabolites was determined with an original specialized program developed in our laboratory [24] similar to the LCModel software package [25], assuming that the spectrum of a mixture of known compounds is a linear combination of analyzed components.

### Primary culture

Primary cultures of rat astrocytes and mixed astrocytes and neurons cultures from hippocampus were prepared from E17 embryonic brains. Astrocyte cultures were seeded onto XFp cell culture miniplate (Seahorse Bioscience) at 5x10^4^ cells/cm^2^ density and grown in Neurobasal 21203, L-glutamine 1 μM, fetal bovine serum 10%, penicillin-streptomycin 50 μg/ml (all reagents from GIBCO Laboratories). In 7 days astrocytes formed a monolayer and allowed for better neuronal attachment to the bottom of the XFp cell culture miniplate. Neurons were seeded onto astrocyte monolayer at 5x10^4^ cells/cm^2^ density and grown in Neurobasal 21203, L-glutamine 1 μM, B-27 supplement, penicillin-streptomycin 50 μg/ml (all reagents from GIBCO Laboratories). For mixed astrocytes and neurons cultures, half of the culture medium volume was exchanged by fresh medium once a week. Astrocytes cultures were used for mitochondrial stress test (MITO STRESS TEST, Seahorse Bioscience) after 17 days in culture, mixed astrocyte and neuron cultures were used after 21–25 days in culture (21–25 days old astrocyte monolayer, 14–19 days old neuronal layer).

### Mitochondrial stress test assay

Mitochondrial stress test assay was performed according to manufacturer’s protocol (MITO STRESS TEST, Seahorse Bioscience). Briefly, cells were pre-incubated with or without 100 μm L-17 for 30 min in thermostat at 37°C in XFp Basal medium (pH = 7.38) with 10 mM glucose and glutamine 1 μM added. Mitochondrial stress test assay was performed on Seahorse XFp (Seahorse Bioscience), allowing for 20 min basal respiration, 20 min with oligomycin, 20 min with FCCP, 20 min with Rothenone/Antimycin A. All respiration values were normalized to protein amount (mg) in each well. Protein measurements were done with standard Bradford assay.

### Statistical analysis

The data are shown as the mean ± SE. The mean values were compared to the control using Mann-Whitney U test and a one-way ANOVA with post hoc least significant difference (LSD) test. The number of initial variables, characterizing the relative number of brain metabolites, was reduced with a partial least squares discriminant analysis (PLS-DA). The correlations of the PLS-DA axes with the metabolites were analyzed by Pearson’s method. Mitochondrial stress test data were analyzed using repeated measurements ANOVA and two-way ANOVA.

## Results

### L-17 significantly reduces rat body temperature

**Fig 2:**
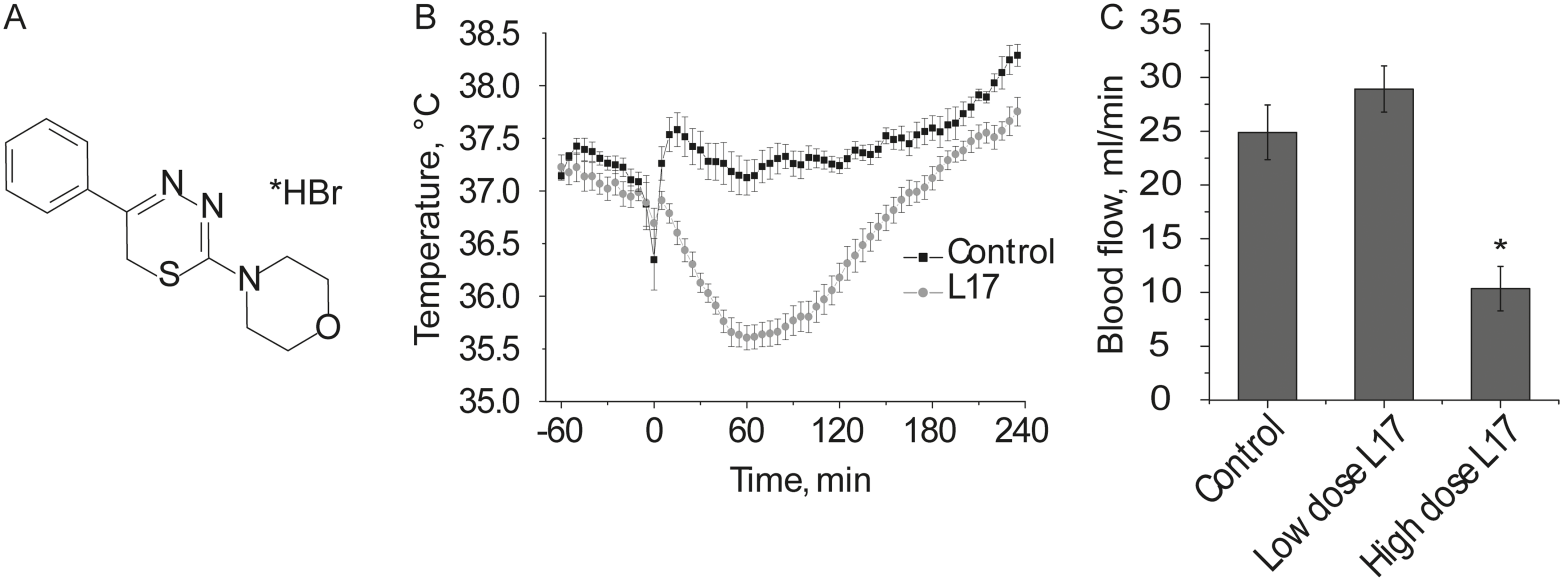
**A** Structural formula of L-17. **B** Changes in body temperature after L-17 intraperitoneal injection 190 mg/kg and **(C)** blood flow velocity. Mann-Whitney U test significant difference from control group ***-p<0.001 n = 8 in (B) and One-way ANOVA * *p*<0.05 significant difference from control group in (C), followed by Post-hoc LSD test.

L-17 reduces mouse body temperature (S1 Fig). We examined whether L-17 can reduce rat body temperature. Injection of L-17 intraperitoneally (190 mg/kg) led to a significant reduction in rat body temperature (Fig 2B, S1 Table). Two minutes after injection of L-17 intraperitoneally caused animals to fall into a sleep-like state with almost no movement. Rat body temperature started to drop within 10 minutes of injection, the effect lasted for 160 min. Lowest mean temperature in rats was 35.6±0.3°C (mean±SD). Rat body temperature fluctuates during twenty-four hours, which we have also observed with implanted temperature data loggers for several days prior to the injection of L-17. Rat body temperature varied throughout the day and night between 36.5 and 38.5°C, however never rat body temperature dropped to the levels observed in L-17 induced hypothermia. L-17 190 mg/kg did not significantly affect blood flow velocity in carotid arteries (Fig 2C, S1 Table). To assess if a higher dose of L-17 may cause toxicity and affect brain metabolites balance we also studied a four times higher dose of L-17 (760 mg/kg). A higher dose of L-17 significantly reduced blood flow velocity in carotid arteries (Fig 2C), indicating a shift towards a pathophysiological state in the brain. Further, we test both doses of L-17 effect onto levels of brain metabolites.

### L-17 effects on brain metabolism in vivo

Altered brain energy turnover inevitably affects balance of most abundant brain metabolites [24]. Several brain metabolites were measured *in vivo* in rat cortex using magnetic resonance spectroscopy. Low dose of L-17 (190 mg/kg) did not induce any significant changes in brain metabolites levels (Table 1), indicating an unchanged energy metabolism. High dose of L-17 (760 mg/kg) showed significant reduction in N-acetylaspartate (NAA) (p<0.01) and a significant increase in taurine (Tau) (p<0.05), indicating neurotoxicity which may be also due to a change in energy metabolism.

**Table 1:** Levels of brain cortex metabolites in control and experimental rats, expressed as the percentage of the total number of molecules detected with ^1^H MRS. NAA, N-acetylaspartate; GABA, gamma-aminobutyric acid; Ala, alanine; Ast, aspartate; Cho, choline; Cr, creatine; PCr, phosphocreatine; Glu, glutamic acid; Gln, glutamine; mIno, myo-inositol; Tau, taurine; Gly, glycine; Lac, lactate; PEA, phosphorylethanolamine.

	NAA %	GABA %	Ala %	Ast %	Cho %	Cr %
Control (N = 7)	16.02±0.45	5.52±0.40	5.25±2.17	0.24±0.09	0.94±0.20	9.98±0.35
L17 low dose (N = 7)	16.30±0.63	6.56±1.65	6.62±2.57	0.51±0.36	0.79±0.29	11.06±0.87
L17 high dose (N = 5)	13.94±0.27^#^	3.54±1.18	3.73±1.13	1.03±0.94	0.64±0.27	9.91±0.49
	Glu+Gln %	mIno %	Tau %	Gly %	Lac %	PEA %
Control (N = 7)	13.18±0.54	1.54±0.84	4.44±0.49	20.80±4.03	6.64±1.45	15.46±1.35
L17 low dose (N = 7)	14.23±2.12	6.34±2.32	4.76±0.44	13.48±4.34	5.48±1.46	13.86±2.85
L17 high dose (N = 5)	16.33±2.79	1.87±1.83	6.14±0.53*	22.83±6.93	4.01±1.55	16.04±4.70

*–significant difference from the control group (*p*<0.05, One-way ANOVA, Post-hoc LSD test)

^#^–significant difference from the control group (*p*<0.01, One-way ANOVA, Post-hoc LSD test)

**Fig 3:**
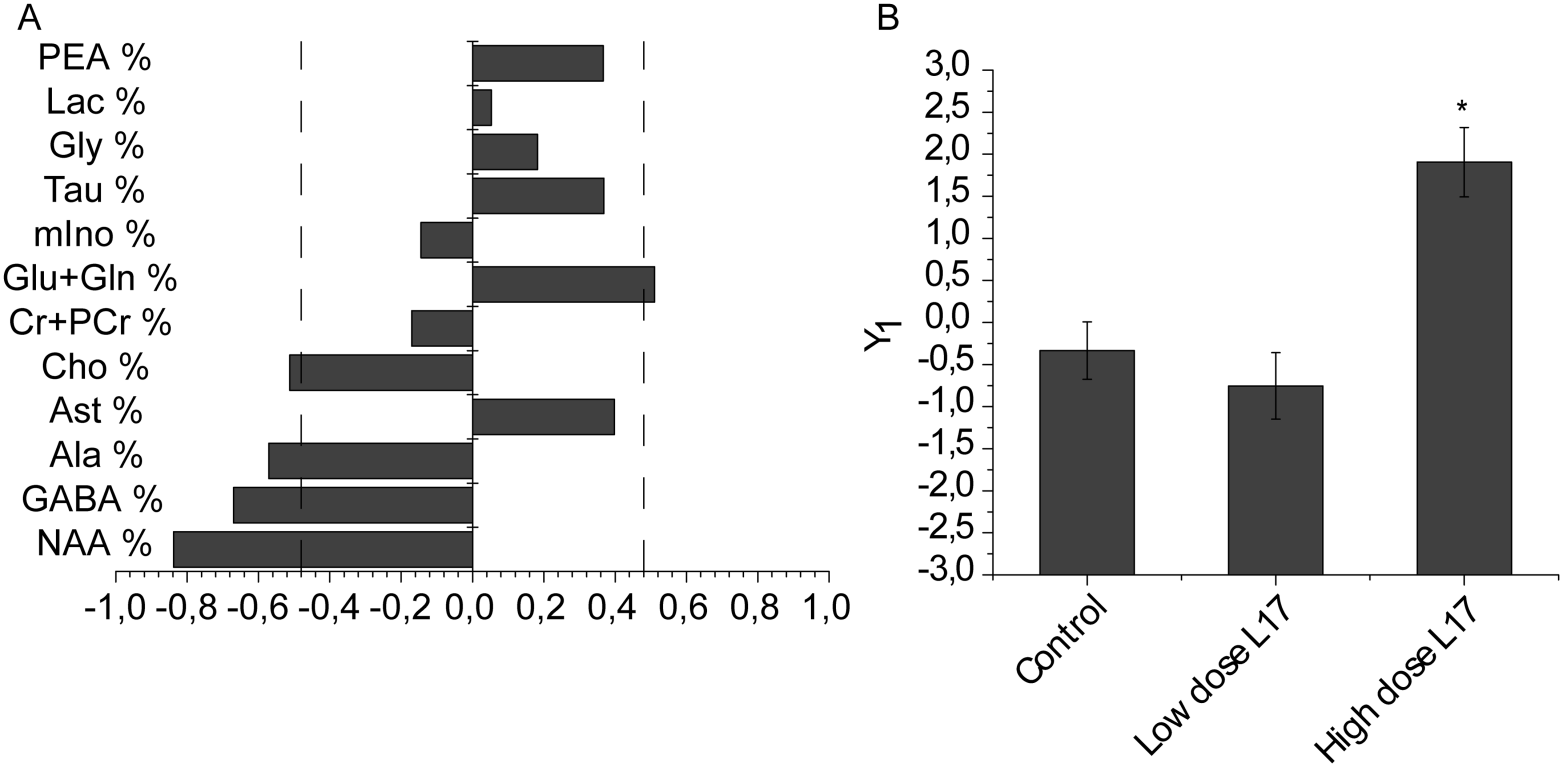
Correlations of individual metabolites with the integral characteristics of metabolic patterns (the values of the Y_1_) in rat brain cortex (A); values of the Y_1_ in rat brain cortex (B). PEA—phosphorylethanolamine; Lac—lactate; Gly—glycine; Tau—taurine; Ins—myo-inositol; Glu—glutamic acid; Gln—glutamine; Cr—creatine; PCr—Phosphocreatine; Cho—choline; Asp—aspartate; Ala—alanine; GABA—gamma-aminobutyric acid; NAA—N-acetylaspartate.

The between-group differences in the metabolomics patterns were detected using multivariate statistical analysis, applied as a standard for the MRS data [26] The PLS-DA showed Y_1_ axis characterizing the variation of statistically linked variables (Fig 3A, S1 Table). This axis was negatively correlated with the levels of N-acetylaspartate (NAA) and choline (Cho), i.e. with the brain metabolites reflecting the viability of neurons and cytoplasmic membrane turnover [27]. In addition, this axis is positively correlated with the levels of glutamic acid and glutamine (Glu+Gln), and negatively with the levels of gamma-aminobutyric acid (GABA). Since Glx are excitatory neurotransmitters, and GABA is an inhibitory one, the values of this axis reflect the balance of excitatory and inhibitory neurotransmitters. The values of the Y_1_ axis are significantly increased in the high dose L-17 group, as compared to control and low dose L-17 groups (*p*<0.05 and *p*<0.005 respectively) (Fig 3B).

### L-17 does not affect mitochondrial function in primary cell culture preparation

**Fig 4:**
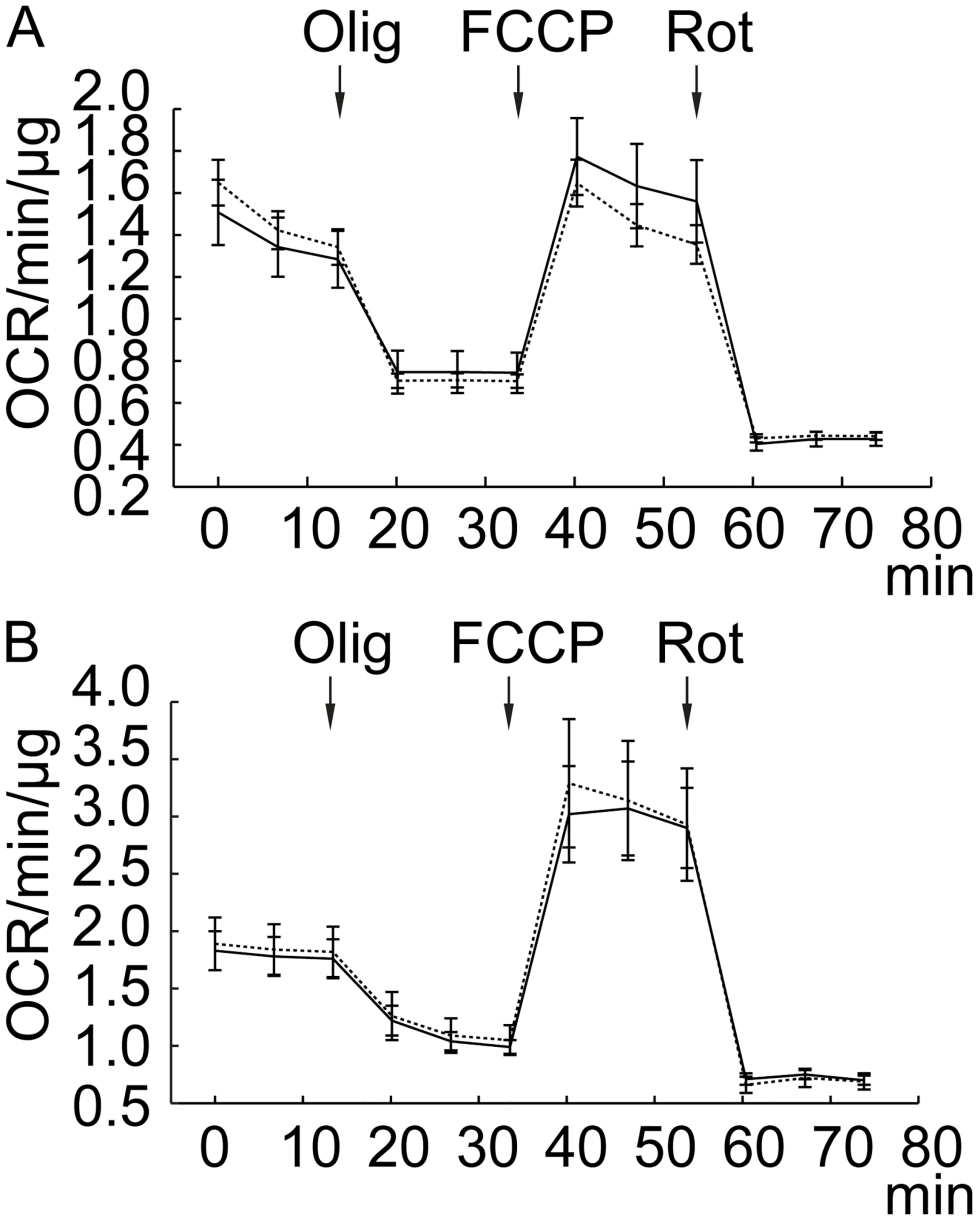
Respiration (OCR) is measured under basal conditions and in response to mitochondrial inhibitors (Oligomycin (Olig), FCCP, Rotenone/Antimycine A (Rot)) in (A) mixed neuron and astrocyte culture (n = 9) and in (B) astrocyte culture (n = 9). Dashed line—control, solid line—L-17 pre-incubation. No significant differences in basal respiration or in mitochondrial stress test assessment were observed in L-17 (100 μM, pre-treated 30 min) cell cultures compared with control.

The reduction in body temperature may occur due to a reduced cellular metabolism, as it occurs for instance with 2-DG treatment—a known cellular ATP depleter [14]. We did not observe any significant changes in brain metabolites with the low dose 190 mg/kg *in vivo*. To exclude the possibility of L-17 effect *in vitro* on cellular metabolism we have measured cellular metabolic rate on the Seahorse bioscience XFp platform—a common measurement of mitochondrial function *in vitro*. For this purpose, we employed Seahorse mitochondrial stress test assay (Seahorse Bioscience). The standard mitochondrial stress test assay allows to measure oxygen consumption rate of the primary culture, which is almost all attributed to the oxygen consumption in oxidative phosphorylation. Two types of rat primary cell culture were used—mixed astrocyte and neuron culture as well as pure astrocyte culture (Fig 4A and 4B, S1 Table). Pre-incubation of either cell culture with L-17 100 μM for 30 min did not affect respiration rates under basal conditions. Apart from basal respiration, we followed the standard mitochondria stress test assay to assess other parameters of mitochondrial function: ATP-linked respiration (blocked with oligomycin), Maximal Respiration (treatment with FCCP), non-mitochondrial respiration (treatment with Rotenone/Antimycin A). We observed no differences in these parameters with L-17 treatment. Leading us to conclude that L-17 reduction in body temperature is not due to a reduction in cellular metabolism and may be attributed to other mechanism.

## Discussion

Previously L-17 compound was shown to benefit recovery after experimentally induced cardiac infarction and pancreatitis in rats [20–22]. Apart from these properties, we have confirmed that L-17 acutely reduces body temperature in rats. Regulation of body temperature depends among others on cellular metabolism. Therefore, in this study we were looking for a link between L-17 induced body temperature decrease and changes in brain metabolism. We found no significant influence of L-17 compound in low dose 190 mg/kg onto a balance of rat brain metabolites, but a significant reduction of body temperature. A similar level of brain metabolites compared to control group indicates physiological brain function under L-17 190 mg/kg treatment, despite induced hypothermia.

A four times higher dose of L-17 (760 mg/kg) showed significant increase in taurine (Tau) (p<0.05) and a significant reduction in N-acetylaspartate (NAA) (p<0.01), which is probably indicative of neuronal toxicity produced by this dose of L-17 [28]. In addition, injection of L-17 in a high dose (760 mg/kg) significantly influenced rat cerebral cortex metabolomic patterns, which were analyzed by multivariate statistics—PLS DA method. The Y_1_ axis, which included the NAA and Cho with a negative sign, reflected a decrease in the viability of nerve cells and cytoplasmic membrane turnover. The Y_1_ axis has maximal values in the group of rats with high dose of L-17, but not in the group with low dose of L17, which did not differ from control. Therewith Y_1_ axis included with a negative sign GABA and with positive sign Glu+Gln, reflecting prevalence of excitatory neurotransmitters over inhibitory ones. Excessive extrasynaptic Glu may lead to excitotoxicity in the brain and neurodegeneration [29]. From this, we can conclude that the high dose of L-17 along with a decrease in carotid arteries blood flow, leads to a decrease in the viability of nerve cells and cytoplasmic membrane turnover and to a balance shift of excitatory and inhibitory neurotransmitters towards excitatory ones. As yet, we do not know whether the L-17 compound is capable of passing through the blood brain barrier and cellular plasma membrane and whether its effect onto brain metabolism in high dose is direct or indirect.

Changes in brain metabolic demand tightly regulate cerebral blood flow [30]. Chemical factors like partial pressure of ambient arterial oxygen and carbon dioxide, pH are products of metabolic activity and can trigger vasodilation or vasoconstriction of arteries leading to a change in blood flow. We observed no changes in blood flow velocity in carotid arteries with 190 mg/kg treatment and a reduced blood flow velocity in carotid arteries with 760 mg/kg dose treatment. Observed reduction in carotid blood flow velocity may be an indication of a reduced brain metabolism with the high L-17 dose. Unchanged blood flow in L-17 190 mg/kg treatment velocity is suggestive of a physiological balance in brain metabolism.

Our *in vitro* studies on primary mixed astrocyte and mixed neuron and astrocyte cultures have shown no effect of L-17 pre-incubation onto basal respiratory rate, ATP-linked respiration or Maximal Respiration. These results support the idea, that L-17 is not inhibiting oxidative phosphorylation and thus has little or no effect onto cellular energy metabolism. Previously on isolated mitochondria preparation treated with similar by structure to L-17 compounds a reduction of mitochondrial function was observed [31], which may be due to the absence of cellular plasma membrane barrier or due to the dissolvent used—DMSO, which is capable of reducing mitochondrial function by itself [32].

Interestingly in a study on mice, the body temperature reduction was much more substantial 8°C (140 mg/kg, 60 min treatment S1 Fig). The temperature effect differences between mice and rats may be due to a reduced hypothermic response to L-17 with increasing body mass. It remains to be investigated whether the hypothermic response observed in rodents may be observed in humans.

As yet, a mechanism by which L-17 reduces the body temperature is unknown. However, we can rule out a direct effect onto cellular metabolism, at least in highest non-toxic dose (190 mg/kg). It is possible that body temperature reduction is due to a direct or indirect L-17 effect onto CNS or peripheral thermoregulatory centers. L-17 was shown to reduce stereotyped chewing behavior induced by apomorphine (non-selective D2-like and D1-like receptors agonist) in mice and reduced serotonin induced spasm of isolated guinea pig ileum muscle, which may indicate possible effects of L-17 on dopaminergic neurotransmission and 5-HT serotonin receptors [33].

Several toxic chemicals cause small rodents to have a regulated hypothermic response, which is thought to be a rodent-specific adaption serving to attenuate the acute toxic insult [34, 35]. Further studies are needed to uncover the mechanism by which L-17 has a hypothermia inducing effect. In the present study, we propose L-17 central action, which may involve the action through the 5-HT receptors or dopamine receptors. To conclude L-17 apart from its anti-inflammatory effects in pancreatitis and cardiac infarction, after further investigations, may have a potential use for induction of mild therapeutic hypothermia.

## Supporting information

S1 Fig Changes in mouse body temperature induced by L-17. Mice BALB/c were injected intraperitoneally with L-17 (140 mg/kg) in NaCl 0.9% or same volume of NaCl 0.9% (control), three mice per group. Temperature measurements were performed using intraperitoneally implanted thermosensitive logger.
(TIF)

S1 Table Individual experiments data file. (PDF)
