# Supplementary figures and images for: Effects of a compound from the group of substituted thiadiazines with hypothermia inducing properties on brain metabolism in rats, a study in vivo and in vitro
Source: PLoS One. 2017 Jul 5;12(7):e0180739. doi: 10.1371/journal.pone.0180739 (PMC5498073; doi:10.1371/journal.pone.0180739)

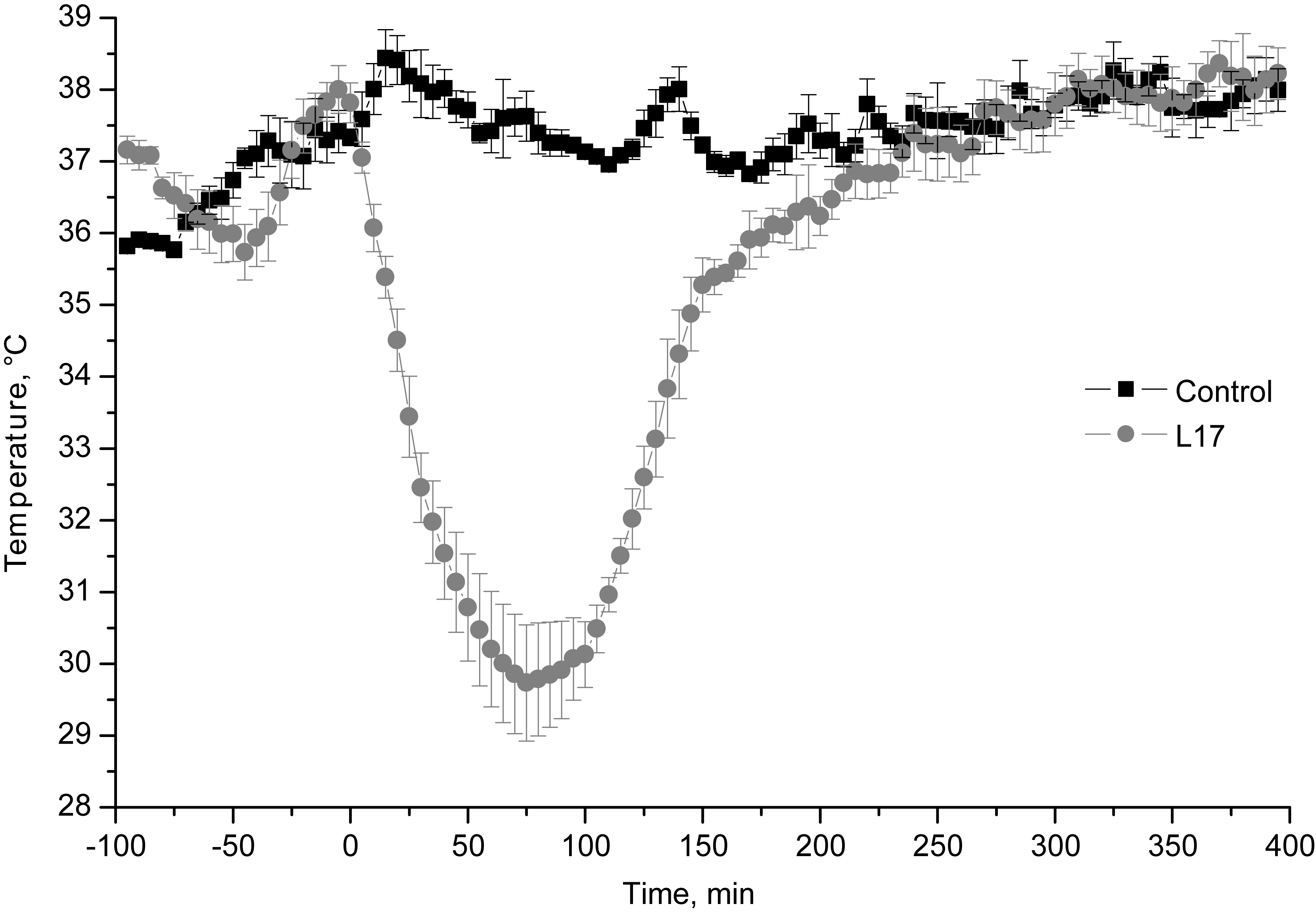

Supplement: S1 Fig — Mice BALB/c were injected intraperitoneally with L-17 (140 mg/kg) in NaCl 0.9% or same volume of NaCl 0.9% (control), three mice per group. Temperature measurements were performed using intraperitoneally implanted thermosensitive logger. (TIF) [file pone.0180739.s001.tif]
