# Supplementary material for: Effects of a compound from the group of substituted thiadiazines with hypothermia inducing properties on brain metabolism in rats, a study in vivo and in vitro
Source: PLoS One. 2017 Jul 5;12(7):e0180739. doi: 10.1371/journal.pone.0180739 (PMC5498073; doi:10.1371/journal.pone.0180739)
Supplement: S1 Table — (PDF) [file pone.0180739.s002.pdf]

Fig 2C Fig 3

Rat	Treatment, mg/kg	right carotide artery blood flow velocity ml/min	left carotide artery blood flow velocity ml/min	sum of blood flow velocities in carotide arteries ml/min	NAA %	GABA %	Ala %	Ast %	Cho %	Cr+PCr %	Glu+Gln %	mIno %	Tau %	Gly %	Lac %	PEA %
1	0	10,7513406	7,0840575	17,8353981	16,49247023	6,384694199	4,263905324	0,097579774	0,311262939	10,24066645	12,12933124	1,999641105	6,297285876	21,00189619	4,788107176	15,99315949
2	0	10,9772091	9,2893554	20,2665645	17,46330433	7,152922073	3,963789227	0,101195618	1,746703053	10,29881144	11,87322699	0,046087152	2,504787671	31,34485251	2,793273629	10,71104631
3	0	11,04538032	14,28145992	25,32684024	16,38793893	4,596484392	4,21303327	0,092009492	0,698208084	10,41944905	15,17045619	1,731280645	3,445739828	14,4881307	9,138748906	19,61852051
4	0	16,19846748	19,47889944	35,67736692	14,97861502	5,344766064	4,283492042	0,093071134	1,300940413	10,12822769	15,1082733	0,334464109	4,824981871	27,34918283	2,786214291	13,46777124
5	0	9,0840204	8,541936	17,6259564	15,42280958	5,723042301	0,829178359	0,708213341	1,233751082	9,012277642	13,4885206	0,385767882	4,187492708	27,83830293	9,517705717	11,65293785
6	0	15,07733838	14,4802242	29,55756258	14,20466884	4,023578908	1,346749894	0,451500114	0,282379504	8,520373806	11,97977864	0,040287702	4,046172193	23,5454933	12,73559349	18,82342361
7	0	13,90200084	14,23875024	28,14075108	17,17194799	5,403279832	17,85828997	0,111499601	1,011572098	11,27072837	12,53512467	6,215988033	5,755214786	0,018353844	4,721709844	17,92629096
8	190	14,11390656	17,58817476	31,70208132	18,85916549	13,89245687	16,06913873	0,116219553	2,314651996	10,40413889	7,252684466	1,546455899	5,915510333	22,73141921	0,839507723	0,058650836
9	190	17,86907304	17,60295888	35,47203192	18,21156481	0,349612443	0,614823317	2,677017059	0,003404734	14,97079544	21,61540302	17,04302419	5,434403764	0,539291977	10,22746312	8,313196123
10	190	16,90646256	18,274815	35,18127756	15,53621008	9,925277621	16,6807093	0,102525828	0,981624067	9,825084134	13,72524439	6,171180233	3,528443386	0,016909473	0,022545964	23,48424552
11	190	13,66956162	12,7471968	26,41675842	16,24941208	3,752363467	1,474368782	0,105116421	0,078577594	13,52819066	21,66663833	10,34721475	6,452807166	3,67529698	5,089410347	17,58060342
12	190	10,91232324	10,5213654	21,43368864	15,96664243	5,016447505	3,026108023	0,084275786	0,609231568	10,55419025	14,13811578	0,973040068	4,073274177	22,44539545	6,940397968	16,172881
13	190	16,7019489	13,1907204	29,8926693	14,26994554	6,281700146	3,524237168	0,361685939	1,030709241	8,744762252	11,93333956	0,047000035	4,351866453	25,54582044	9,154826077	14,75410714
14	190	11,52175752	11,0305962	22,55235372	15,01883966	6,70104131	4,952843032	0,093396383	0,544861968	9,410580796	9,281899676	8,266773491	3,583899717	19,42212107	6,062633763	16,66110913
15	760	4,82619384	3,24265032	8,06884416	14,31522534	4,903046153	6,287568769	0,079744709	0,85605301	8,654909347	11,65038348	0,036315347	6,016490962	26,69042521	5,506788802	15,00304886
16	760	3,6221094	3,09973716	6,72184656	13,26514148	0,031598432	2,970850259	3,859640211	0,003361535	10,29436592	24,36717229	7,363704681	4,774426037	4,474054176	0,894168408	27,70151658
17	760	8,63967546	7,46433792	16,10401338	13,7549675	4,351485438	4,658612698	0,084750726	0,451864432	9,700486083	13,8386306	0,038568692	6,428338367	22,36959013	7,618697031	16,7040083
18	760	7,45119648	3,18351384	10,63471032	14,43489224	4,885601713	1,009542065	0,083954195	1,238585842	10,97204316	15,44624079	0,038221502	7,347941173	37,79364953	2,008625509	4,740702287

Fig 4A

Control	basal OCR/µg			Oligomycin			FCCP			Rot		
well 1	1,499716473	1,323578481	1,256183856	0,708288455	0,714807269	0,733499393	1,365504357	1,266419671	1,224487542	0,429724395	0,421838562	0,431243321
well 2	1,199157337	1,027628208	0,963395269	0,520680709	0,534821274	0,541799825	1,330242193	1,094652533	1,000648737	0,29809348	0,323266793	0,324724656
well 3	1,364846487	1,189557766	1,115688927	0,619693543	0,620179383	0,626048359	1,378538922	1,239328628	1,176965459	0,403165461	0,416637091	0,411871573
well 4	1,928513554	1,666573768	1,567387648	0,815188884	0,823014501	0,813531996	1,927621989	1,755131511	1,680924755	0,439093717	0,479222827	0,463224301
well 5	1,98859555	1,674851668	1,582576829	0,776107923	0,782866465	0,76279045	2,104063398	1,848195123	1,702861461	0,449835975	0,480606009	0,477898795
well 6	1,716428628	1,527718361	1,467099374	0,784365067	0,801398081	0,803676804	1,824517049	1,651221413	1,539347755	0,46693653	0,477139203	0,486541176
well 7	2,125862938	1,817494703	1,692061328	0,814772522	0,793840445	0,775744264	1,894997233	1,553717534	1,458793794	0,514207405	0,520846653	0,497485191
well 8	1,71136398	1,435928286	1,352695486	0,695961482	0,685368668	0,667863922	1,835360214	1,606788399	1,442488551	0,443246848	0,450412027	0,452710695
well 9	1,307154225	1,14121325	1,083362677	0,610607711	0,612375412	0,605596016	1,162661419	1,002476102	0,964219743	0,432604511	0,419642406	0,422043753
L-17 100uM	basal OCR/µg			Oligomycin			FCCP			Rot		
well 1	1,002928488	0,86953284	0,820934033	0,487069726	0,480700126	0,486887694	1,095643668	0,972392083	0,903027402	0,318730632	0,325774319	0,337763887
well 2	1,422972038	1,286227274	1,226809166	0,657138849	0,660513507	0,659454208	1,686558734	1,409257173	1,332971732	0,414809127	0,425029434	0,436041594
well 3	0,637293804	0,557478315	0,542497923	0,344295141	0,359352017	0,376033628	0,781601063	0,662402336	0,625834045	0,207415353	0,216266281	0,235926597
well 4	1,822434984	1,649632883	1,59279297	0,788299125	0,788321501	0,79171785	2,187510827	2,063021548	1,952478355	0,444888038	0,490829326	0,481513858
well 5	2,059668199	1,809416047	1,731064058	0,822893888	0,832718002	0,822461167	2,193125295	2,257030789	2,197422528	0,479250159	0,514382446	0,50745418
well 6	1,242171619	1,09178215	1,03684344	0,657904516	0,649355363	0,649315108	1,520388798	1,321890495	1,286907793	0,356278425	0,3749323	0,354956261
well 7	1,770767046	1,55309215	1,473468236	1,453342475	1,431384425	1,411818205	2,421357184	2,518709829	2,439839933	0,511812959	0,538173945	0,542477384
well 8	1,805813563	1,633063447	1,56005003	0,773845107	0,775656122	0,760387031	2,031224262	1,753071352	1,631205948	0,478797085	0,501075454	0,490007479
well 9	1,80647726	1,629108561	1,572305359	0,733829917	0,743985395	0,73064479	2,040232664	1,738439901	1,668353729	0,42547789	0,459388498	0,462084236

Fig 4B

Control	basal OCR/µg			Oligomycin			FCCP			Rot		
well 1	1,541724785	1,550215594	1,489655642	1,479263515	1,499342984	1,511475924	2,989879398	2,682791361	2,594549648	0,552260684	0,602641063	0,559925775
well 2	1,241239736	1,199690545	1,211773921	0,593218935	0,609806431	0,637783692	1,973034266	1,829480815	1,764797686	0,457541803	0,542135947	0,492840983
well 3	2,645325874	2,627707411	2,634562363	1,436515252	1,303459224	1,274176176	5,84973835	5,40066106	5,281456759	0,858966005	0,892139403	0,870142632
well 4	2,238195013	2,098923871	2,057276116	1,051047341	1,038102398	1,063404916	2,655519445	2,685543348	2,436447991	0,712352251	0,837938564	0,784043816
well 5	1,521061103	1,461244192	1,411176702	0,89042306	0,813539319	0,857892115	2,338632235	2,342096192	2,172230791	0,617402614	0,64759884	0,585887882
well 6	3,017758654	2,876072794	2,841002391	2,365160296	1,725623268	1,567274562	5,348381979	5,538069647	5,16171745	1,090637704	1,172905486	1,124541898
well 7	2,423377066	2,343915281	2,375627716	2,054197711	1,574281786	1,292303778	5,108236237	4,377368882	3,741129674	0,791703796	0,808860359	0,802143617
well 8	1,116760622	1,118515624	1,081785758	0,646605422	0,584638849	0,541870498	1,3520633	1,368517644	1,316668086	0,418536988	0,441749352	0,466713291
well 9	1,283358727	1,245856381	1,257149522	0,796080199	0,680696366	0,659833132	2,018814588	2,056038988	1,858045687	0,480760438	0,514053016	0,513754602
L-17 100uM	basal OCR/µg			Oligomycin			FCCP			Rot		
well 1	2,669371573	2,619627248	2,581771138	1,338522184	1,3118739	1,26499895	4,862010414	4,570006422	4,342575303	0,914520216	0,95585758	0,875562265
well 2	1,490419151	1,426911473	1,435952492	1,074761499	1,0361068	1,017395544	2,23051747	2,136341676	2,136663172	0,551141854	0,61770968	0,562626805
well 3	1,40123902	1,422262677	1,400411337	0,778538341	0,79051864	0,76887594	2,297077363	2,175640515	2,177026795	0,557185859	0,572932587	0,568479684
well 4	1,428202547	1,35798302	1,358453841	0,930895535	0,828689632	0,848660863	1,827952212	1,894209745	1,900820591	0,750221288	0,732013213	0,661113376
well 5	1,469762452	1,395633002	1,376553718	0,875838128	0,801187724	0,854701642	1,921795105	2,191984559	2,128521536	0,647272855	0,722270487	0,664824005
well 6	1,656382721	1,610017014	1,565229741	1,286584003	1,059632745	0,959676868	2,748493928	2,939551873	2,645798567	0,657417928	0,715906483	0,621629756
well 7	2,2649631	2,171149395	2,206427573	1,719712122	1,162317885	1,066364954	3,755521458	4,132590494	3,736170361	0,84150733	0,840784039	0,838806515
well 8	2,23779465	2,199096724	2,190936572	1,794891311	1,329098756	1,129818899	4,509320988	4,493149068	4,100346497	0,755710514	0,805349071	0,777039358

Fig 2B

190 mg/kg L-17 Rat#1 T °C	190 mg/kg L-17 Rat#2 T °C	Control Rat#3 T °C	Control Rat#4 T °C	Control Rat#5 T °C	190 mg/kg L-17 Rat#6 T °C	Control Rat#7 T °C	190 mg/kg L-17 Rat#8 T °C
37,076	36,839	36,985	36,75	37,105	36,506	36,718	36,753
37,05	37,009	36,834	36,807	37,074	36,639	36,68	36,838
37,044	36,877	36,815	36,641	37,105	36,791	36,737	36,74
37,05	36,946	36,639	36,546	37,13	36,702	36,849	36,74
37,044	36,896	36,708	36,546	37,161	36,753	36,399	36,766
37,134	36,958	36,884	36,348	37,334	36,791	36,323	36,864
37,134	37,078	36,922	36,616	37,309	36,873	36,373	36,877
37,012	37,204	36,834	36,692	37,334	36,886	36,687	36,838
36,902	37,204	36,702	36,807	37,352	36,506	36,756	36,851
36,819	37,216	36,651	36,909	37,284	36,379	36,862	36,864
36,819	37,16	36,677	37,017	37,352	36,493	36,943	36,753
36,947	37,172	36,79	36,979	37,074	36,538	36,881	36,714
36,864	37,003	36,821	36,756	37,031	36,69	36,799	36,675
36,819	36,782	36,973	36,762	36,957	36,468	37,031	36,669
36,838	36,814	37,004	36,781	36,914	36,772	37,037	36,552
36,986	36,826	37,042	36,941	36,889	36,721	36,724	36,552
36,941	36,927	36,998	36,883	36,901	36,785	36,837	36,773
36,819	36,839	36,859	36,813	36,926	36,924	36,492	36,766
36,766	36,908	36,847	36,788	37,073	36,924	36,449	36,669
36,824	36,965	36,897	36,718	37,159	36,956	36,48	36,609
36,888	36,94	36,897	36,699	37,258	37,006	36,634	36,446
36,914	36,757	36,847	36,659	37,351	37,057	36,722	36,42
36,991	36,707	36,803	36,691	37,468	36,823	36,797	36,348
37,036	36,732	36,804	36,678	37,462	36,709	36,834	36,394
37,049	36,606	36,917	36,486	37,456	36,861	36,703	36,459
36,997	36,644	36,728	36,569	37,487	36,74	36,565	36,459
36,817	36,675	36,766	36,582	37,561	36,772	36,502	36,407
36,817	36,782	36,911	36,754	37,221	36,778	36,477	36,453
36,817	36,864	36,949	36,818	37,295	36,747	36,59	36,231
36,965	36,77	36,911	36,907	37,258	36,658	36,847	36,173
37,004	36,682	36,943	36,844	37,351	36,519	36,978	36,218
36,978	36,568	37,037	36,856	37,283	36,538	37,022	36,107
37,004	36,612	37,113	36,907	36,925	36,639	36,847	36,153
37,075	36,499	37,251	37,016	36,647	36,652	36,822	36,12
37,049	36,543	37,144	36,62	36,548	36,677	36,834	36,199
36,875	36,531	37,144	36,423	36,703	36,797	36,847	36,466
36,817	36,55	37,056	36,544	36,808	36,816	36,822	36,7
36,779	36,631	36,873	36,557	36,746	36,519	36,866	36,98
36,689	36,669	36,917	36,435	36,585	36,468	36,866	36,876
36,824	36,682	36,791	36,461	36,647	36,075	36,859	36,96
36,862	36,908	36,615	36,518	36,696	36,221	36,878	36,589
36,978	36,927	36,583	36,544	36,715	36,36	36,747	36,602
36,94	36,826	36,646	36,582	36,777	36,538	36,766	36,524
36,978	36,933	36,791	36,557	36,845	36,696	36,697	36,524
37,03	36,688	36,741	36,397	36,943	36,652	37,022	36,537
37,023	36,449	36,766	36,423	36,956	36,519	37,054	36,524
37,041	36,474	36,741	36,531	36,956	36,62	36,916	36,518
37,112	36,682	36,741	36,544	36,937	36,709	36,916	36,321
37,067	36,675	36,596	36,372	36,937	36,715	36,903	36,38
36,932	36,524	36,898	36,281	37,061	36,348	36,647	36,38
36,887	36,473	36,936	36,121	37,122	36,354	36,49	36,295
36,919	36,567	36,834	36,287	36,734	36,265	36,553	36,347
36,938	36,384	36,897	36,338	36,74	36,272	36,703	36,002
37,009	36,661	36,935	36,287	36,913	36,272	36,878	35,865
36,848	36,624	36,973	36,287	36,863	36,221	36,722	36,08
36,816	36,743	36,992	36,185	37,005	36,278	36,703	36,074
36,874	36,8	37,08	36,147	37,129	36,31	36,903	36,295
36,983	36,8	36,847	36,109	37,042	36,329	36,866	36,38
36,655	36,863	36,821	36,077	37,042	36,278	36,866	36,464
36,501	36,8	36,689	36,23	37,067	36,519	36,69	36,575
36,507	36,661	36,708	36,223	36,943	36,683	36,809	36,464
36,52	36,384	36,714	36,223	36,82	36,702	36,459	36,523
36,604	36,353	36,714	36,185	36,863	36,493	36,571	36,575
36,629	36,504	36,79	36,236	36,727	36,417	36,54	36,295

36,687	36,447	36,821	36,198	36,653	36,367	36,59	36,334
36,713	36,573	36,727	36,077	36,493	36,506	36,69	36,334
36,752	36,668	36,727	36,529	36,345	36,55	36,734	36,321
36,803	36,605	36,708	36,389	36,456	36,658	36,647	36,497
36,726	36,624	36,677	36,357	36,622	36,664	36,709	36,282
36,649	36,624	36,815	36,338	36,684	36,538	36,665	36,158
36,604	36,624	36,834	36,16	36,9	36,506	36,709	36,282
36,552	36,73	36,777	36,089	37,061	36,367	36,978	36,38
36,687	36,642	36,765	36,16	37,104	36,183	37,035	36,464
36,777	36,693	36,872	36,325	37,092	36,012	36,822	36,627
36,834	36,718	36,62	36,415	36,979	36,1	36,797	36,477
36,686	36,693	36,569	36,555	36,942	36,289	36,64	36,354
36,654	36,756	36,626	36,408	36,967	36,358	36,608	36,354
36,718	36,63	36,601	36,434	37,257	36,219	36,633	36,432
36,75	36,624	36,752	36,67	37,399	36,023	36,889	36,126
36,911	36,705	36,823	36,472	37,164	36,441	36,977	36,158
37,001	36,743	36,917	36,485	37,066	36,479	37,046	36,23
37,2	36,8	36,98	36,447	37,121	36,871	37,04	36,393
37,232	36,8	37,182	36,345	37,17	36,821	37,034	36,484
37,232	36,73	37,113	36,147	37,245	36,909	37,046	36,634
37,162	36,8	37,289	36,332	37,319	36,929	37,14	36,354
37,2	36,837	37,194	36,593	37,195	36,814	37,265	36,607
37,162	36,9	37,194	36,906	37,183	36,713	37,034	36,458
37,11	36,944	37,125	36,925	37,195	36,814	36,846	36,419
37,149	36,787	37,094	36,893	37,177	36,814	36,927	36,49
37,11	36,781	36,974	36,938	37,158	36,929	37,046	36,751
37,187	36,768	36,974	37,02	37,282	36,979	37,027	36,953
37,129	36,787	37,006	37,129	37,294	36,954	37,002	37,154
37,335	36,888	37,056	37,084	37,096	37,093	37,052	36,861
37,541	36,888	37,182	37,091	37,146	37,081	37,115	37,024
37,599	37,045	37,276	37,275	37,164	37,062	37,234	36,927
37,477	37,265	37,516	37,454	37,269	36,922	37,29	36,588
37,393	37,29	37,743	37,722	37,528	36,929	37,259	36,523
37,393	37,171	37,95	37,741	37,306	36,967	37,221	36,575
37,612	37,02	37,843	37,511	37,374	36,922	37,159	36,64
37,258	37,007	37,547	37,518	37,269	36,897	37,209	36,725
37,046	36,907	37,44	37,333	37,485	36,922	37,259	36,432
37,046	36,875	37,358	37,084	37,615	37,005	37,29	36,471
37,117	36,894	37,377	36,906	37,579	37,112	37,146	36,282
37,117	36,718	37,346	36,867	37,53	37,044	37,034	36,549
37,11	36,787	37,157	37,161	37,561	37,152	37,008	36,588
37,2	36,85	37,131	35,552	37,585	37,165	37,084	35,129
**37,007**	**35,238**	**35,361**	**37,433**	**37,215**	**37,152**	**35,95**	**36,347**
37,065	36,54	37,382	37,911	37,561	36,994	37,046	36,367
36,885	36,591	37,703	37,873	37,826	36,829	37,378	36,321
36,988	36,609	37,785	37,484	37,962	36,506	37,528	36,139
36,898	36,458	37,621	37,032	38,06	36,303	37,534	36,106
36,692	36,351	37,533	37,223	37,653	36,088	37,447	35,937
36,339	36,276	37,792	37,287	37,505	35,885	37,365	35,839
36,217	36,15	37,514	37,274	37,48	35,727	37,196	35,748
35,998	36,144	37,508	37,261	37,517	35,657	37,234	35,67
35,882	36,031	37,596	37,369	37,499	35,587	37,146	35,487
35,786	36,169	37,458	37,083	37,561	35,505	36,758	35,474
35,741	36,15	37,521	36,917	37,715	35,486	36,72	35,474
35,728	36,207	37,395	37,076	37,678	35,41	36,758	35,533
35,753	36,282	37,445	37,172	37,678	35,308	36,877	35,618
35,914	36,377	37,489	37,274	37,906	35,302	37,071	35,676
35,991	36,383	37,502	37,095	37,838	35,289	37,234	35,715
36,011	36,408	37,527	37,312	37,857	35,302	37,422	35,781
36,159	36,653	37,596	37,325	37,727	35,308	37,265	35,878
36,326	36,616	37,426	37,236	37,746	35,308	37,303	35,937
36,422	36,565	37,527	37,191	37,832	35,308	37,159	36,093
36,409	36,823	37,558	37,236	37,832	35,334	37,084	36,184
36,409	36,861	37,596	37,287	37,604	35,448	37,077	36,23
36,487	36,981	37,703	37,357	37,567	35,492	37,096	36,23
36,506	36,962	37,489	37,223	37,598	35,632	37,096	36,295
36,642	36,911	37,483	37,216	37,431	35,803	36,958	36,367

36,977	36,911	37,628	37,223	37,585	35,945	37,196	36,202
37,002	36,968	37,514	37,229	37,69	36,046	37,303	36,202
36,996	36,974	37,571	37,18	37,567	36,141	37,472	36,294
36,989	37,003	37,464	37,078	37,48	36,28	37,534	36,391
37,176	37,034	37,47	37,556	37,487	36,331	37,259	36,443
38,421	37,091	37,489	37,524	37,561	38,469	37,716	36,508
38,222	37,135	37,439	37,333	37,703	38,647	37,541	38,304
38,325	37,191	37,464	37,333	37,677	38,533	37,814	38,33
38,53	37,869	36,992	37,31	38,139	38,754	38,333	38,018
38,646	37,775	37,761	37,929	38,491	38,653	38,233	37,731
38,678	37,41	38,152	37,884	38,479	38,545	37,939	37,842
38,64	37,183	38,461	38,184	38,547	38,368	38,458	38,031
38,575	37,183	38,429	38,063	38,454	38,368	38,246	37,64
38,659	37,668	38,001	37,986	38,534	38,394	38,189	37,64
38,575	37,819	37,976	37,954	38,584	38,381	38,127	37,829
38,781	37,901	38,057	37,903	38,571	38,552	38,058	37,555
38,556	37,857	37,95	37,98	38,787	38,311	37,964	37,516
38,601	37,485	38,391	37,916	38,584	38,387	38,058	37,816
38,71	38,096	38,486	37,986	38,547	38,381	37,995	37,803
38,774	38,265	38,681	38,241	38,466	38,697	38,058	37,77
38,517	38,02	38,832	37,731	38,411	38,482	37,851	37,9
38,434	37,643	38,637	37,738	38,417	38,159	36,981	37,933
38,395	37,812	38,555	37,738	38,368	38,14	37,663	38,109
38,46	37,913	38,593	37,922	38,257	38,242	37,983	38,272
38,492	37,938	38,605	37,597	38,38	38,292	37,839	38,031
38,318	37,75	38,612	37,75	38,584	38,09	37,951	37,816
38,492	37,857	38,435	37,75	38,701	38,128	37,995	37,881
38,505	37,743	38,253	37,776	38,608	37,602	37,908	37,998
38,479	37,825	38,561	37,859	38,571	37,767	38,127	37,972
38,447	37,995	38,618	37,827	38,522	37,754	38,064	38,057
38,652	38,02	38,662	37,731	38,25	38,102	38,077	38,031
38,729	37,989	38,599	37,763	37,956	38,172	38,077	37,946
38,742	37,951	38,631	37,897	38,332	38,292	37,87	37,848
38,492	37,938	38,379	37,578	38,468	38,273	37,914	37,705
38,395	37,592	38,461	37,739	38,388	38,026	37,488	37,419
38,068	37,334	38,435	37,828	38,301	38,077	37,676	37,406
38,055	37,397	38,415	37,777	37,906	37,583	37,432	37,438
37,997	37,416	38,182	37,573	37,956	37,431	37,263	37,315
37,547	37,064	37,899	37,236	37,851	37,361	37,206	37,282
37,483	37,045	37,861	37,382	37,82	37,52	37,044	37,21
37,477	36,888	37,653	37,032	37,77	37,57	37,231	37,21
37,444	36,787	37,792	36,923	37,598	37,45	37,263	37,262
37,425	36,447	37,527	37,197	37,573	36,943	37,137	37,087
37,284	36,397	37,319	36,77	37,45	36,778	37,137	37,139
37,072	36,504	37,225	36,649	37,375	36,69	36,95	37,139
37,136	36,397	37,124	36,63	37,375	36,709	36,705	37,178
37,136	36,46	37,08	36,77	37,375	37,032	36,649	37,184
37,091	36,705	37,073	36,891	37,363	37,038	36,605	37,048
37,117	36,655	37,067	37,032	37,332	37,038	36,586	36,794
37,104	36,598	37,143	36,987	37,431	37,051	36,58	36,794
37,072	36,554	37,036	36,891	37,45	36,354	36,605	37,015
37,174	36,498	37,206	37,019	37,4	36,272	36,624	36,976
37,091	36,554	37,067	36,891	37,425	36,392	36,624	36,956
37,065	36,617	36,803	36,917	37,363	36,671	36,586	36,943
37,136	36,592	36,645	36,961	37,295	36,633	36,574	36,976
37,149	36,579	36,859	37,025	37,196	36,645	36,586	36,989
37,162	36,529	37,01	37,178	37,153	36,626	36,58	37,022
37,033	36,592	37,017	37,07	37,036	36,772	36,586	36,807
36,988	36,617	37,08	37,012	37,098	36,677	36,63	36,807
37,043	36,611	37,143	37,108	37,214	36,696	36,461	36,781
37,024	36,447	37,225	37,172	37,164	36,652	36,474	36,903
37,062	36,422	37,168	36,783	37,121	36,678	36,442	36,929
37,101	36,649	37,193	36,764	37,164	36,893	36,687	36,929
37,082	36,419	37,124	36,878	37,294	36,963	36,43	36,942
37,127	36,407	37,179	36,923	37,177	37,02	36,524	37,053
37,268	36,432	37,167	36,949	36,843	37,026	36,474	37,066
37,377	36,407	37,116	36,674	36,454	36,918	36,549	37,098

37,397	36,778	37,217	36,764	36,825	36,975	36,63	37,163
37,435	36,627	37,192	36,7	36,967	36,253	36,712	37,183
37,307	36,545	37,217	36,764	37,103	36,773	36,806	37,046
37,268	36,532	37,223	36,776	37,121	36,937	36,962	37,001
37,172	36,627	37,204	36,923	36,769	36,937	36,768	37,059
37,242	36,47	37,33	37,083	36,936	36,963	36,862	37,066
37,229	36,488	37,154	36,904	37,028	36,95	37,031	36,805
37,21	36,897	36,896	36,821	36,874	37,108	37,056	36,818
37,339	37,155	36,883	36,821	36,825	37,14	37,206	36,779
37,21	37,313	36,99	36,961	36,541	37,013	37,031	36,89
37,172	37,432	37,053	36,936	36,732	36,963	36,993	36,864
37,332	36,941	37,154	36,961	36,812	37,216	36,981	36,949
37,21	37,294	37,236	36,949	36,775	36,937	36,981	36,981
37,21	37,338	37,299	36,745	36,849	37,001	36,856	37,059
37,319	37,048	37,122	36,553	36,849	37,051	36,856	37,15
37,082	37,092	37,015	36,541	36,837	37,013	36,887	36,936
37,21	37,08	37,041	36,579	36,942	37,001	37,018	36,805
37,474	36,935	37,015	36,732	36,967	37,083	37,094	36,844
37,538	36,941	36,971	36,725	37,053	37,039	37,125	36,656
37,268	36,778	36,94	36,706	37,146	37,058	37,05	36,909
36,894	36,778	36,965	36,483	37,245	37,026	36,912	36,883
36,919	36,86	36,959	36,509	37,146	37,066	36,831	36,83
37,08	36,778	36,996	36,553	37,183	37,104	36,937	36,804
37,112	36,872	36,978	36,635	37,109	37,174	37,012	36,791
37,106	36,962	36,965	36,628	37,294	37,212	36,887	36,895
37,196	36,968	37,066	36,526	37,177	37,212	36,818	36,908
37,266	36,949	37,192	36,481	36,954	37,288	36,843	36,941
37,382	36,968	37,085	36,507	37,109	37,332	37,012	37,006
37,421	36,993	37,122	36,507	37,059	37,383	37,031	36,986
37,434	36,999	37,066	36,526	36,843	37,503	36,887	37,123
37,446	36,955	37,154	36,622	36,874	37,326	36,962	37,149
37,363	37,031	37,104	36,679	36,936	37,313	37,062	37,149
37,356	37,037	37,167	36,762	37,115	37,421	37,044	37,162
37,35	37,069	37,129	36,513	36,991	37,231	36,981	37,182
37,241	37,169	36,87	36,456	37,14	37,136	36,925	37,175
37,254	37,157	36,845	36,373	37,084	37,136	36,899	37,149
37,222	36,974	36,959	36,258	37,004	37,167	36,806	37,071
37,414	36,716	36,978	36,233	37,078	37,288	36,674	37,006
37,53	36,408	36,952	36,348	37,041	37,566	36,699	36,921
37,716	36,547	36,996	36,411	37,103	37,478	36,693	36,934
37,53	36,635	36,688	36,379	37,115	37,224	36,693	36,993
37,479	36,754	36,7	36,526	36,979	37,034	36,768	36,732
37,157	36,609	36,883	36,386	36,942	37,041	36,705	36,628
37,356	36,716	37,034	36,405	36,991	36,952	36,705	36,706
37,408	36,729	37,072	36,469	37,103	36,977	36,762	36,817
37,453	36,798	37,104	36,469	37,183	37,091	36,793	36,882
37,543	36,704	37,122	36,507	37,17	37,186	36,743	36,908
37,479	36,823	37,154	36,481	37,201	37,433	36,755	36,602
37,596	36,924	37,053	36,539	37,158	37,497	36,749	36,915
37,467	36,999	37,141	36,539	36,899	37,37	36,95	36,764
37,416	37,018	36,984	36,577	36,782	37,37	37,018	36,484
37,416	37,069	36,814	36,513	36,757	37,357	36,937	36,503
37,57	37,073	36,801	36,507	36,732	37,452	36,968	36,542
37,512	37,168	36,845	36,367	36,769	37,547	37,006	36,731
37,48	37,105	36,738	36,316	36,757	37,465	37,006	36,588
37,403	37,237	36,65	36,552	36,954	37,503	36,78	36,64
37,409	37,13	36,656	36,622	36,967	37,433	36,806	36,757
37,352	37,18	36,644	36,622	36,664	37,357	36,937	36,757
37,422	37,023	36,663	36,737	36,559	37,288	36,962	36,803
37,512	36,897	36,669	36,819	36,751	37,262	36,755	36,803
37,384	36,878	36,763	36,813	36,837	37,275	36,874	36,894
37,409	36,778	36,896	36,877	36,936	37,345	36,831	36,855
37,319	36,727	36,984	36,851	36,924	37,452	36,856	36,965
37,319	36,998	37,066	36,851	36,726	37,535	36,818	37,063
37,358	37,092	37,217	36,839	36,782	37,345	36,956	37,148
37,461	37,042	37,248	36,921	36,979	37,345	37,018	37,271
37,654	36,74	36,858	36,921	37,004	37,345	37,062	37,278

37,885	36,941	37,129	36,775	36,979	37,56	37,125	37,219
38,065	37,187	37,028	36,705	36,954	37,737	37,363	37,271
37,956	37,25	37,381	36,934	36,837	37,788	37,025	37,245
37,853	37,583	37,261	37,234	36,708	37,566	37,012	37,271
37,744	37,721	37,639	37,323	36,843	37,345	37,15	37,245
37,692	37,709	37,91	37,559	37,059	37,332	37,244	37,35
37,679	38,042	37,872	37,444	36,979	37,345	37,231	37,389
37,506	38,42	37,778	36,902	36,849	37,446	36,968	37,376
37,808	38,407	37,488	36,972	36,874	37,693	36,987	37,174
37,935	38,432	37,488	37,126	36,941	37,446	37,062	37,148
37,704	38,25	37,469	37,119	37,077	37,516	37,087	37,122
37,479	38,181	37,45	37,087	36,935	37,395	37,156	37,135
37,594	38,017	37,532	36,902	36,749	37,509	37,231	37,07
37,607	37,962	37,62	36,864	36,719	37,623	37,112	37,174
37,607	37,691	37,721	36,526	36,861	37,68	37,15	37,258
37,536	37,616	37,274	36,692	36,762	37,788	37,1	37,232
37,543	37,49	37,192	36,679	36,953	37,927	37,044	37,109
37,472	37,452	37,337	36,552	36,644	37,946	37,006	37,018
37,543	37,484	37,167	36,921	36,7	37,718	37,018	37,011
37,543	37,421	37,261	37,074	36,756	37,642	37,075	37,018
37,472	37,603	37,444	37,132	36,756	37,68	37,119	37,018
37,543	37,547	37,393	37,228	36,848	37,383	37,432	37,063
37,607	37,509	37,431	36,921	36,953	37,313	37,595	37,161
37,543	37,201	37,33	37,011	37,132	37,459	37,688	37,135
37,581	37,081	37,362	37,062	37,114	37,427	37,482	37,174
37,556	37,169	37,406	36,826	37,114	37,3	37,388	36,978
37,556	37,012	37,419	36,89	37,293	37,326	37,219	36,803
37,62	37,132	37,526	37,132	37,268	37,541	37,325	36,731
37,62	37,358	37,607	36,89	37,305	37,079	37,4	36,816
37,453	37,478	37,626	36,851	37,336	37,047	37,419	36,803
37,459	37,641	37,607	36,96	37,36	37,224	37,425	36,933
37,305	37,748	37,488	37,023	37,373	37,566	37,482	37,057
37,266	37,761	37,494	37,106	37,453	37,427	37,413	37,278
37,395	37,691	37,595	37,17	37,336	37,497	37,3	37,356
37,35	37,641	37,582	36,839	37,151	37,516	37,432	37,258
37,318	37,496	37,324	36,8	37,114	37,357	37,457	37,245
37,356	37,371	37,311	37,049	37,163	37,37	37,375	37,271
37,336	37,295	37,532	37,081	37,362	37,262	37,325	37,297
37,143	37,169	37,752	37,043	37,386	37,503	37,375	37,356
37,323	37,213	37,639	37,1	37,399	37,731	37,424	37,343
37,156	37,339	37,639	36,89	37,553	37,554	37,43	37,232
36,956	37,522	37,677	36,73	37,59	37,383	37,468	37,297
37,053	37,276	37,715	37,132	37,547	37,953	37,474	36,972
37,207	37,283	37,607	37,1	37,399	37,706	37,618	37,076
37,207	37,314	37,437	36,768	37,522	37,516	37,499	37,083
37,239	37,434	37,45	37,106	37,516	37,421	37,286	37,083
37,278	37,446	37,45	37,183	37,448	37,566	37,286	37,174
37,098	37,534	37,437	37,285	37,319	37,623	37,374	37,174
37,169	37,509	37,475	37,285	37,331	37,63	37,312	37,174
37,406	37,534	37,519	37,31	37,454	37,427	37,299	36,985
37,432	37,522	37,607	37,298	37,473	37,592	37,474	36,939
37,664	37,641	37,557	37,087	37,541	38,559	37,549	36,939
37,734	37,591	37,469	36,768	37,64	38,375	37,543	38,067
37,387	37,471	37,532	36,775	37,652	38,324	37,781	38,145
37,58	37,602	37,639	37,8	37,991	38,362	37,925	38,008
37,792	37,401	37,959	37,596	38,3	38,502	38,176	38,126
38,043	37,394	38,028	37,232	38,374	38,54	38,125	37,982
38,255	37,463	38,198	37,576	38,072	38,464	38,163	37,82
38,261	37,413	38,129	37,8	38,226	38,299	38,113	37,702
37,664	37,224	38,299	38,125	38,121	38,166	38,019	37,716
37,484	37,344	38,538	37,825	38,355	38,039	38,169	37,364
37,831	37,96	38,456	37,946	38,355	38,191	38,063	37,865
37,786	38,111	37,719	37,653	38,399	38,039	38,244	37,768
37,336	38,086	36,749	37,831	38,516	38,191	38,157	37,709
37,811	38,237	38,009	37,761	38,676	38,286	38,244	37,911
38,145	38,181	38,526	37,953	38,442	38,267	37,875	38,008
37,477	38,017	38,469	38,144	38,3	38,109	38,075	37,611

37,978	37,828	38,274	38,035	38,084	38,084	38,088	37,598
37,458	37,765	38,217	38,125	37,911	38,267	38,207	37,768
38,582	37,835	38,249	38,048	37,843	38,179	38,207	38,008
38,454	38,193	38,28	37,87	38,127	37,991	38,407	38,113
38,537	38,08	38,267	37,921	38,479	37,946	38,457	37,852
38,345	38,262	38,091	37,576	38,547	38,326	38,357	37,93
38,345	38,218	38,16	37,423	38,559	38,244	38,113	37,93
38,313	38,03	38,351	37,506	38,645	37,965	38,088	37,781
37,773	37,872	38,401	37,921	38,559	38,155	37,862	37,702
38,28	37,872	38,218	37,353	38,034	38,257	38,006	37,364
38,338	37,545	38,162	37,27	37,954	38,041	37,556	37,344
38,306	37,847	38,124	37,27	37,917	37,908	37,888	37,839
38,39	38,137	38,023	37,583	38,343	37,851	37,831	38,015
38,1	37,948	37,929	37,946	38,152	37,668	37,593	38,008
38,197	37,589	38,08	37,87	37,96	37,573	37,412	37,787
38,004	37,413	37,935	37,863	37,664	37,357	37,405	37,273
37,831	37,369	37,639	37,895	37,547	37,142	37,424	37,051
37,773	37,344	37,828	37,876	37,269	36,99	37,412	37,012
37,631	37,589	37,463	37,557	37,047	36,92	37,368	36,986
37,529	37,344	37,607	37,417	37,201	36,781	37,286	36,947
37,458	37,357	37,677	37,494	37,362	36,787	37,074	36,993
37,413	37,363	36,845	37,149	37,374	36,87	36,873	36,986
37,496	37,212	37,374	37,309	37,269	36,844	36,898	36,915
37,355	37,067	37,356	37,086	37,096	36,8	36,704	36,863
37,381	36,797	37,154	36,945	36,936	36,787	36,66	36,778
37,374	36,753	37,028	36,818	36,911	36,813	36,698	36,706
37,278	36,627	36,952	36,85	36,967	36,502	36,767	36,765
37,349	36,545	36,82	36,811	37,066	36,413	36,829	36,778
37,278	36,564	36,845	36,811	37,127	36,42	36,854	36,791
37,143	36,539	36,845	36,862	37,207	36,464	36,842	36,817
37,095	36,532	36,845	36,811	37,28	36,521	36,898	36,869
37,121	36,702	36,858	36,913	37,28	36,583	36,911	36,866
37,224	36,784	36,984	36,971	37,293	36,697	37,071	36,84
37,217	36,765	37,009	36,899	37,33	36,766	37,203	36,599
37,326	36,715	36,978	36,689	37,348	36,88	37,084	36,528
37,224	36,765	37,039	36,746	37,256	36,937	37,058	36,599
37,153	36,614	36,693	36,861	37,212	36,925	37,077	36,586
37,095	36,161	36,579	36,899	37,157	36,855	37,002	36,606
37,069	36,218	36,642	36,931	37,237	36,811	37,008	36,586
36,986	36,218	36,869	37,007	37,015	36,868	37,177	36,58
36,986	36,218	36,712	37,09	36,749	36,95	37,084	36,639
36,986	36,262	36,775	37,16	36,719	36,975	37,033	36,463
37,011	36,52	36,812	37,294	36,737	36,95	37,015	36,599
36,921	36,325	36,831	37,282	36,885	36,925	37,04	36,775
36,954	36,004	36,951	37,148	36,941	36,728	37,071	36,658
36,979	36,073	37,033	37,084	36,965	36,792	37,021	36,743
37,024	36,123	36,964	37,097	37,107	36,792	36,983	36,86
37,121	36,092	36,982	37,122	36,898	36,684	36,99	36,919
37,191	36,136	37,039	37,129	36,873	36,804	36,858	36,938
36,966	36,256	37,115	36,848	36,774	36,817	36,977	37,01
36,889	36,218	37,108	36,918	36,786	36,906	36,827	37,049
36,812	36,167	36,951	36,746	36,558	36,88	36,827	36,586
36,684	36,564	36,775	36,625	36,428	36,925	36,689	36,58
36,703	36,753	36,932	36,561	36,731	36,817	36,664	36,704
36,812	36,74	36,976	36,561	36,786	36,754	36,739	36,827
36,864	36,457	36,976	36,612	36,873	36,804	36,99	36,573
36,934	36,595	37,19	36,574	36,861	36,937	37,127	36,756
36,754	36,595	37,14	36,638	36,885	36,785	37,102	36,899
36,714	36,564	36,982	36,644	36,952	36,69	37,19	36,873
36,676	36,558	37,045	36,669	37,155	36,811	37,253	36,652
36,689	36,614	37,127	36,714	37,155	36,792	37,052	36,704
36,676	36,671	37,14	36,803	37,118	36,83	37,065	36,853
36,766	36,885	37,178	36,899	36,896	37,026	36,727	36,814
36,824	36,822	37,216	37,09	36,723	37,115	36,676	36,938
36,888	36,935	37,216	37,078	36,711	37,184	36,626	36,958
36,907	36,872	37,039	37,078	36,594	37,172	36,689	37,036
36,933	36,885	36,995	37,122	36,433	36,608	36,658	36,99

37,139	36,488	36,857	37,282	36,427	36,399	36,689	36,997
37,222	36,224	37,008	37,097	36,489	36,412	36,708	36,99
37,139	36,186	36,989	36,848	36,297	36,45	36,789	37,003

190 mg/kg L-17 Rat#9 T °C	Control Rat#10 T °C	Control Rat#11 T °C	Control Rat#12 T °C	190 mg/kg L-17 Rat#13 T °C	190 mg/kg L-17 Rat#14 T °C	190 mg/kg L-17 Rat#15 T °C	190 mg/kg L-17 Rat#16 T °C
36,761	36,892	36,859	37,415	36,787	37,058	37,132	36,664
36,729	36,948	36,853	37,396	36,681	37,027	37,125	36,708
36,583	36,986	36,789	37,51	36,693	37,09	37,075	36,791
36,551	36,948	36,776	37,624	36,687	37,039	37,037	36,766
36,564	36,917	36,679	37,504	36,681	37,071	36,834	36,778
36,564	36,955	36,615	37,478	36,656	37,121	36,841	36,848
36,551	36,811	36,532	37,415	36,737	36,957	36,929	36,886
36,57	36,679	36,493	37,32	36,768	36,799	36,815	36,55
36,462	36,648	36,519	37,275	36,681	36,768	36,992	36,29
36,513	36,679	36,455	37,294	36,712	36,761	37,011	36,823
36,609	36,704	36,487	37,104	36,737	36,793	37,049	36,943
36,787	36,729	36,57	37,097	36,631	36,787	37,1	36,873
36,825	36,779	36,628	37,104	36,631	36,907	37,163	36,962
36,825	36,905	36,416	37,142	36,557	36,976	37,113	37,007
36,539	36,961	36,352	37,104	36,594	36,989	37,062	36,924
36,398	37,036	36,468	36,71	36,644	37,039	37,062	36,791
36,239	37,068	36,403	36,85	36,65	36,957	37,087	36,804
36,265	36,742	36,519	36,596	36,644	37,076	37,17	36,79
36,284	36,666	36,545	36,507	36,476	37,114	37,049	36,916
36,309	36,811	36,628	36,481	36,555	36,987	36,834	37,043
36,328	36,911	36,756	36,507	36,704	36,905	36,605	37,1
36,328	36,905	36,795	36,678	36,63	36,905	36,523	36,91
36,335	36,903	36,808	36,875	36,605	36,931	36,573	36,974
36,36	36,689	36,833	36,92	36,617	36,394	36,681	36,942
36,443	36,702	36,731	36,951	36,661	36,451	36,744	36,999
36,539	36,765	36,75	36,939	36,76	36,526	36,864	37,031
36,679	36,677	36,737	36,691	36,81	36,514	36,953	37,05
36,723	36,595	36,776	36,666	36,804	36,463	36,966	37,062
36,742	36,658	36,84	36,697	36,767	36,552	36,902	36,986
36,793	36,664	36,833	36,92	36,854	36,501	36,909	37,043
36,825	36,633	36,782	36,939	36,711	36,539	36,928	37,018
36,99	36,677	36,711	36,958	36,723	36,419	37,004	37,062
37,016	36,771	36,808	37,085	36,736	36,457	37,143	37,031
37,131	36,827	36,789	37,129	36,785	36,457	37,099	37,037
36,971	36,846	36,711	37,218	36,729	36,457	36,978	37,1
37,016	36,727	36,769	37,18	36,748	36,571	37,01	36,847
36,959	36,646	36,814	37,18	36,835	36,583	37,004	36,974
36,99	36,646	36,833	37,25	36,91	36,691	37,035	37,081
36,908	36,677	36,827	37,155	36,791	36,501	37,111	37,119
36,959	36,796	36,731	37,104	36,773	36,621	37,143	37,1
36,99	36,852	36,679	37,097	36,791	36,539	37,162	37,196
37,029	36,921	36,705	37,097	36,779	36,583	37,099	37,227
37,003	36,884	36,801	37,104	36,76	36,691	37,004	37,253
36,99	36,909	36,84	37,072	36,68	36,735	36,94	36,859
36,978	36,928	36,763	37,097	36,711	36,716	36,953	36,713
36,946	36,871	36,789	36,939	36,729	36,582	36,871	36,649
36,959	36,758	36,782	36,907	36,798	36,651	36,852	36,788
36,971	36,677	36,795	36,945	36,784	36,822	36,814	36,915
36,717	36,677	36,724	37,085	36,821	36,854	36,825	36,979
36,685	36,664	36,609	37,085	36,79	36,961	36,844	37,099
36,933	36,288	36,581	36,932	36,803	36,986	36,838	37,112
36,99	36,169	36,613	36,793	37,163	37,068	36,863	36,947
37,111	36,257	36,498	36,735	37,002	37,011	36,888	36,94
37,105	36,301	36,434	36,761	37,281	36,854	36,831	36,82
37,175	36,37	36,485	36,869	37,294	36,86	36,711	36,82
37,188	36,088	36,556	36,735	37,194	36,734	36,679	36,833
37,111	36,037	36,331	36,748	36,933	36,443	36,654	36,814
37,213	36,207	36,331	36,805	36,846	36,38	36,717	36,788
37,188	36,257	36,395	36,881	36,859	36,557	36,604	36,953
37,118	36,037	36,369	36,812	36,678	36,443	36,572	36,959
36,819	36,244	36,402	36,812	36,566	36,456	36,616	36,934
36,793	36,263	36,299	36,862	36,647	36,595	36,762	36,928
36,685	36,376	36,292	36,881	36,566	36,67	36,667	36,915
36,8	36,257	36,305	36,913	36,554	36,696	36,521	36,972

36,831	36,407	36,318	37,002	36,554	36,929	36,585	37,08
36,857	36,558	36,35	37,047	36,616	36,974	36,597	36,991
36,946	36,752	36,382	37,097	36,66	37,062	36,711	36,991
36,742	36,714	36,607	37,104	36,579	36,986	36,774	36,959
36,768	36,658	36,639	37,104	36,548	37,182	36,857	36,985
36,819	36,495	36,652	37,212	36,405	36,929	36,774	37,105
36,812	36,382	36,69	37,18	36,554	36,866	36,755	37,207
36,793	36,263	36,761	37,116	36,554	36,569	36,724	37,194
36,793	36,276	36,768	37,428	36,579	36,393	36,717	37,169
36,787	36,457	36,774	37,167	36,548	36,43	36,768	37,08
36,761	36,646	36,806	37,155	36,66	36,386	36,85	37,118
36,78	36,501	36,768	37,059	36,708	36,538	36,888	37,207
36,723	36,689	36,851	36,824	36,752	36,62	36,845	37,181
36,755	36,677	36,812	36,71	36,783	36,677	37,004	37,036
36,729	36,608	36,742	36,601	36,783	36,664	37,117	37,048
36,806	36,702	36,652	36,531	36,795	36,525	37,117	37,061
36,729	36,777	36,652	36,55	36,87	36,55	37,073	36,991
36,749	36,664	36,639	36,664	36,839	36,557	37,162	37,023
36,819	36,495	36,613	36,69	36,72	36,658	37,219	37,029
36,793	36,376	36,8	36,918	36,702	36,797	37,174	37,124
36,761	36,451	36,832	37,083	36,77	36,557	37,174	37,169
36,787	36,451	36,735	37,083	36,795	36,582	37,193	37,22
36,761	36,545	36,832	36,88	36,851	36,449	37,187	37,264
36,8	36,689	36,832	36,887	36,932	36,38	37,035	37,061
36,812	36,646	36,857	37,071	36,975	36,715	36,896	37,036
36,908	36,664	36,857	37,071	36,963	36,765	36,839	37,099
36,717	36,689	36,639	37,102	36,745	36,715	36,978	37,181
36,564	36,765	36,665	37,102	36,857	36,651	37,004	37,518
36,551	36,771	36,665	36,918	36,919	36,607	37,136	37,778
36,729	36,909	36,812	36,994	37,131	36,607	37,244	37,644
36,92	37,047	36,992	37,102	37,33	36,847	37,478	37,657
37,092	37,354	37,198	37,28	37,566	37,011	37,427	37,327
37,226	37,203	37,326	37,331	37,647	37,131	37,725	37,22
37,143	37,297	37,063	37,401	37,516	36,689	37,712	37,105
37,092	37,448	37,146	37,49	37,423	36,753	37,586	37,08
36,959	37,354	37,191	37,382	37,361	36,809	37,503	37,036
37,137	37,222	37,121	37,344	37,361	36,847	37,44	36,985
37,162	37,21	37,076	37,477	37,591	36,955	37,396	37,003
36,971	37,166	37,268	37,464	37,156	36,771	37,383	36,984
36,978	36,84	37,127	37,382	36,951	36,513	37,383	37,022
37,118	36,765	36,992	37,268	36,907	36,727	37,25	37,047
37,258	36,796	36,883	37,115	37,05	36,822	37,396	37,098
**36,959**	**36,533**	**36,169**	**36,88**	**36,011**	**36,651**	**37,01**	**36,406**
36,876	37,241	36,798	37,61	36,907	36,86	37,035	37,18
36,513	37,761	37,209	37,941	36,708	37,068	36,978	36,996
36,157	37,786	37,433	38,074	36,683	36,784	36,883	36,673
35,946	37,786	37,607	38,023	36,553	36,525	36,7	36,463
35,794	37,836	37,433	37,896	36,497	36,569	36,535	36,318
35,768	37,68	37,292	37,915	36,41	36,241	36,352	36,191
35,749	37,629	37,337	37,674	36,322	36,146	36,194	36,134
35,698	37,623	37,222	37,706	36,254	36,121	36,061	35,835
35,373	37,617	37,023	37,814	36,167	36,096	35,966	35,531
34,934	37,636	37,048	37,763	36,111	36,051	35,903	35,505
35,106	37,479	37,337	37,35	35,999	36,039	35,846	35,379
35,049	37,435	37,061	37,407	35,924	36,032	35,795	35,372
35,112	37,341	37,016	37,356	35,868	36,083	35,776	35,391
35,112	37,322	37,048	37,369	35,85	36,058	35,763	35,417
35,1	37,435	37,17	37,439	35,819	36,07	35,738	35,442
35,1	37,285	37,234	37,414	35,806	36,083	35,776	35,436
35,265	37,228	37,311	37,509	35,757	36,134	35,751	35,436
35,303	37,247	37,003	37,503	35,75	36,279	35,763	35,48
35,342	37,128	37,17	37,407	35,819	36,146	35,776	35,544
35,144	37,128	37,433	37,452	35,887	36,026	35,82	35,626
35,221	37,235	37,485	37,337	35,999	36,298	35,846	35,759
35,342	37,166	37,241	37,242	36,179	36,291	35,896	35,842
35,424	37,178	37,337	37,153	36,366	36,336	35,884	36
35,533	37,222	37,414	37,287	36,546	36,437	36,042	36,045

35,634	37,059	37,51	37,331	36,707	36,715	36,194	36,127
35,72	37,366	37,626	37,395	36,719	36,86	36,349	36,184
35,854	37,31	37,363	37,464	36,763	37,125	36,476	36,222
35,981	37,065	37,405	37,739	36,931	37,024	37,799	36,305
36,223	37,153	37,533	37,669	37,067	36,929	37,97	38,111
36,274	37,41	37,597	37,802	37,111	37,056	38,082	38,06
38,263	37,253	37,54	37,967	38,231	38,673	38,114	38,098
38,168	37,103	37,591	38,069	38,206	38,439	38,051	38,181
38,092	38,356	38,201	38,12	38,206	38,42	38,006	38,136
38,321	38,293	38,284	37,618	38,474	38,225	38,051	38,251
38,429	38,262	38,483	38,101	38,567	38,414	38,272	38,175
38,276	38,074	38,425	37,917	38,592	38,471	38,089	38,035
38,168	38,099	38,368	37,796	38,318	38,572	38,38	38,016
38,181	38,149	38,393	37,72	38,088	38,628	38,816	37,991
37,907	38,25	38,438	37,231	38,063	38,439	38,822	38,219
37,748	38,306	38,259	37,65	38,182	38,187	38,595	38,073
37,722	38,588	38,259	37,802	38,113	38,464	38,519	38,111
37,805	38,538	38,214	37,929	38,262	38,496	38,595	38,048
37,856	38,494	38,239	38,24	38,356	38,288	38,595	38,073
37,773	38,412	38,252	37,809	38,219	38,452	38,607	38,06
37,512	38,488	38,271	37,859	38,169	38,37	38,626	38,263
37,977	38,369	38,098	37,898	38,405	38,559	38,664	38,606
38,098	38,369	38,259	38,374	38,567	38,616	38,727	38,745
38,55	38,224	38,413	38,463	38,113	38,591	38,582	38,549
38,575	38,08	38,297	38,279	38,287	38,591	38,316	38,409
38,607	38,174	38,098	38,336	38,269	38,37	38,443	38,441
38,397	38,112	38,098	38,221	38,486	38,338	38,462	38,504
38,308	38,112	38,008	38,215	38,138	38,521	38,626	38,282
38,34	38,074	37,912	38,215	38,144	38,401	38,607	38,32
38,225	37,761	38,098	38,323	38,076	38,199	38,417	38,492
38,251	37,986	38,015	38,215	38,405	37,864	38,468	38,601
38,066	37,711	37,668	38,552	38,169	37,958	38,361	38,607
38,053	37,554	37,585	38,469	37,849	37,99	38,367	38,455
37,784	37,366	37,45	38,082	37,781	38,047	38,221	38,423
37,912	37,279	37,418	38,088	37,912	37,876	38,221	38,246
37,606	37,673	37,429	37,807	37,769	37,769	38,127	38,062
37,772	37,767	37,448	37,909	37,713	37,542	38,101	37,833
37,791	37,773	37,461	37,858	37,626	37,561	38,247	37,643
37,733	37,73	37,435	37,649	37,619	37,548	38,12	37,63
37,784	37,673	37,332	37,414	37,607	37,46	38,006	37,63
37,632	37,617	37,121	37,407	37,532	37,636	37,81	37,586
37,612	37,667	37,018	37,382	37,551	37,605	37,785	37,611
37,434	37,46	36,883	37,395	37,595	37,58	37,659	37,561
37,18	37,435	36,8	37,363	37,221	37,573	37,595	37,573
37,25	37,31	36,954	37,153	37,246	37,542	37,551	37,611
37,25	37,31	36,922	36,994	37,246	37,365	37,602	37,611
37,186	36,909	37,146	36,677	37,315	37,239	37,355	37,624
37,192	36,821	37,018	36,626	37,315	37,264	37,273	37,63
37,224	36,852	37,153	36,588	37,346	37,321	37,228	37,63
37,364	36,852	37,005	36,55	37,327	37,359	37,165	37,624
37,46	36,953	36,902	36,613	37,383	37,112	37,285	37,447
37,549	36,946	36,825	36,664	37,464	37,049	37,134	37,25
37,638	36,934	36,806	36,69	37,52	36,942	37,07	37,25
37,695	36,852	36,864	36,677	37,738	36,847	37,07	37,408
37,746	36,802	36,941	36,677	37,781	36,866	37,045	37,472
37,791	36,809	36,992	36,709	37,738	36,917	36,975	37,573
37,829	36,815	36,992	36,721	37,638	36,904	36,975	37,611
37,422	36,877	37,018	36,785	37,57	36,942	37,07	37,624
37,504	36,834	36,941	36,391	37,601	36,841	37,121	37,609
37,498	36,796	36,896	36,302	37,52	36,961	37,228	37,469
37,587	36,658	36,665	36,398	37,52	36,999	37,247	37,425
37,53	36,658	36,607	36,48	37,42	37,106	37,379	37,393
37,555	36,658	36,703	36,664	37,501	37,176	37,366	37,412
37,6	36,677	36,796	36,693	37,327	37,22	37,505	37,45
37,555	36,664	36,225	36,693	37,315	37,308	37,29	37,216
37,364	36,664	36,238	36,789	37,19	37,359	37,145	37,26
37,377	36,533	36,289	36,871	37,116	37,258	37,138	37,285

37,339	36,689	36,289	36,662	37,19	37,346	37,082	37,139
37,262	36,483	36,128	36,3	37,234	37,378	37,12	36,981
37,224	36,74	36,193	36,376	37,047	37,403	37,006	36,607
37,186	36,871	36,417	36,497	37,097	37,195	37,031	36,695
37,186	36,796	36,167	36,566	37,153	37,075	37,113	37,006
37,167	36,752	36,366	36,624	37,153	37,163	37,132	37,222
37,199	36,815	36,648	36,516	37,172	37,245	36,746	37,406
37,161	36,871	36,591	36,554	37,166	37,365	36,74	37,583
37,091	36,877	36,636	36,585	36,973	37,441	36,987	37,438
37,04	36,915	36,892	36,585	36,973	37,119	37,006	37,621
37,014	36,984	37,014	36,611	37,066	37,157	37,069	37,374
37,014	36,834	36,809	36,611	37,116	37,201	37,031	37,463
36,721	36,865	36,777	36,662	37,134	37,207	37,056	37,609
36,645	36,915	36,591	36,719	37,122	37,207	37,044	37,45
36,664	36,852	36,494	36,878	36,973	37,176	37,113	37,177
36,683	36,796	36,424	36,687	36,805	37,131	37,145	37,038
36,734	36,903	36,36	36,89	37,215	37,056	36,999	36,968
36,721	36,896	36,443	36,903	37,526	36,974	37,056	37,025
36,721	36,946	36,623	37,093	37,545	36,974	37,056	37,07
37,129	36,884	36,77	37,005	37,539	37,024	37,101	37,068
37,148	36,821	36,565	36,941	37,408	36,462	37,101	37,056
37,224	36,871	36,437	37,017	37,277	36,361	37,208	37,246
36,987	36,909	36,449	37,03	37,203	36,43	37,226	37,316
37	36,758	36,494	37,1	37,215	37,062	37,207	37,328
37,063	36,726	36,449	36,928	37,134	37,144	37,162	37,354
37	36,876	36,539	36,662	36,649	37,182	37,169	37,36
37,063	36,92	36,526	36,624	36,438	37,119	37,251	37,411
37,095	36,895	36,578	36,757	36,426	37,201	37,327	37,506
37,184	36,958	36,61	36,865	36,55	37,182	37,339	37,436
37,229	36,939	36,552	36,871	36,544	37,056	37,434	37,474
37,159	36,945	36,488	36,992	36,481	36,734	37,409	37,607
36,904	36,845	36,77	36,598	36,656	36,74	37,245	37,569
36,866	36,826	36,725	36,649	36,649	36,753	37,232	37,404
36,904	36,851	36,796	36,624	36,774	36,797	37,162	37,246
36,866	36,952	36,636	36,611	36,761	36,841	37,067	37,271
36,917	36,97	36,591	36,687	37,29	36,929	37,08	37,436
36,987	37,021	36,841	36,839	37,495	37,011	37,112	37,519
37,191	36,833	36,873	37,093	37,514	37,239	37,131	37,417
36,942	36,964	36,713	37,112	37,439	36,986	37,137	37,462
36,357	37,077	36,745	36,674	37,103	36,607	37,137	37,417
36,325	37,064	36,745	36,687	36,817	36,708	37,156	37,246
36,274	37,033	36,687	36,77	36,718	36,961	37,093	37,354
36,675	37,046	36,7	36,935	37,134	37,15	37,162	37,481
36,688	36,795	36,847	36,897	37,116	37,169	37,213	37,443
36,745	36,864	36,905	36,782	37,166	37,195	37,327	37,519
36,726	36,964	36,854	36,706	37,116	37,22	37,181	37,468
36,93	37,021	36,803	36,731	37,172	37,283	36,922	37,424
36,987	37,064	36,803	36,731	37,34	37,081	36,435	37,455
36,866	36,87	36,88	36,795	37,302	36,961	36,403	37,354
36,739	36,82	36,963	36,941	37,11	36,955	36,435	37,271
36,59	36,795	37,111	37,1	37,072	37,037	36,452	37,221
36,718	36,858	37,124	37,151	36,917	37,15	36,762	37,221
36,724	36,92	37,202	37,195	36,929	37,232	36,699	37,075
36,711	36,908	37,331	37,151	37,103	37,182	36,876	37,062
36,654	36,776	37,363	37,112	37,128	37,169	36,933	37,214
36,711	36,833	37,388	37,195	37,184	37,22	36,876	37,404
36,826	36,845	37,446	37,227	37,184	37,239	36,851	37,474
36,89	36,901	37,446	37,1	37,178	37,321	37,003	37,468
36,877	36,851	37,273	37,252	36,942	37,131	37,041	37,506
36,902	36,864	37,016	37,246	37,022	36,778	37,123	37,5
36,915	36,826	36,856	37,112	37,122	36,753	37,193	37,468
36,953	36,739	36,779	37,093	37,153	37,056	37,18	37,493
37,068	36,958	36,92	37,1	37,159	37,037	37,041	37,347
37,087	36,814	36,868	37,138	37,327	37,138	36,996	37,462
37,036	37,058	36,901	37,201	36,811	37,226	37,117	37,62
37,061	36,889	37,074	37,163	36,761	37,346	37,42	37,677
37,227	36,77	37,388	37,208	37,396	37,403	37,68	37,906

37,272	36,895	37,414	37,259	37,582	37,195	37,926	38,14
37,272	37,234	37,581	37,392	37,613	37,163	37,863	38,337
37,214	37,315	37,575	37,506	37,874	37,46	37,642	38,438
37,443	37,284	37,388	37,633	38,017	37,58	37,566	38,216
37,583	37,597	37,247	37,741	38,204	37,933	37,585	38,254
37,685	37,848	37,575	38,046	37,893	37,737	37,458	38,254
37,583	38,098	38,018	38,313	37,912	37,782	37,484	38,191
38,099	38,098	38,223	38,313	37,974	37,763	37,553	38,001
38,016	38,023	38,39	38,332	37,619	37,889	37,597	37,621
38,207	37,979	38,255	38,408	37,563	37,611	37,61	37,729
38,099	37,685	38,095	38,16	37,433	37,548	37,68	37,691
37,77	37,697	38,037	37,913	37,221	37,567	37,795	37,653
37,706	37,572	37,941	37,798	37,346	37,611	37,909	37,736
37,63	37,447	37,729	37,506	37,489	37,618	37,757	37,717
37,783	37,528	37,613	37,646	37,253	37,573	37,852	37,761
37,814	37,716	37,613	37,913	37,24	37,63	37,782	37,767
37,802	37,848	37,543	38,059	37,253	37,592	37,795	37,799
37,789	37,697	37,536	38,103	37,277	37,592	37,808	37,831
37,789	37,284	37,427	38,078	37,34	37,668	37,498	37,78
37,757	37,29	37,427	37,938	37,253	37,769	37,485	37,704
37,547	36,757	37,337	37,678	37,439	37,782	37,586	37,729
37,432	36,713	37,331	37,582	37,346	37,725	37,447	37,704
37,312	36,864	37,324	37,532	37,396	37,807	37,213	37,723
37,248	37,058	37,17	37,532	37,396	37,75	36,859	37,729
37,203	37,14	36,965	37,506	37,408	37,605	37,194	37,558
37,502	37,221	37,177	37,341	37,327	37,485	37,365	37,317
37,891	37,183	37,023	37,341	37,253	37,472	37,352	37,406
37,802	37,046	37,003	37,1	37,327	37,491	37,567	37,621
37,598	36,983	37,023	37,189	37,34	37,466	37,567	37,482
37,547	37,077	37,145	37,386	37,215	37,479	37,561	37,514
37,471	37,227	37,266	37,601	37,302	37,378	37,314	37,387
37,63	37,102	37,016	37,659	37,141	37,428	37,409	37,482
37,445	36,77	37,555	37,398	37,01	37,479	37,567	37,216
37,394	36,701	37,664	37,176	37,097	37,58	37,618	37,095
37,407	36,858	37,838	37,24	37,134	37,605	37,599	37,323
37,515	36,995	37,838	37,341	37,29	37,769	37,561	37,469
37,617	37,19	37,774	37,532	37,327	37,58	37,624	37,609
37,572	37,234	37,774	37,773	37,396	37,668	37,713	37,621
37,541	37,227	37,684	37,906	37,394	37,794	37,485	37,596
37,445	37,077	37,594	37,582	37,488	37,782	37,484	37,488
37,432	37,277	37,51	37,703	37,488	37,782	37,237	37,596
37,547	37,221	37,457	37,265	37,55	37,946	37,306	37,438
37,649	37,397	37,547	37,335	37,444	37,737	37,42	37,406
37,655	37,409	37,522	37,462	37,345	37,378	37,294	37,621
37,496	37,29	37,432	37,449	37,158	37,283	37,585	37,767
37,566	37,672	37,483	37,57	37,052	37,365	37,743	37,609
37,63	37,447	37,412	37,913	37,276	37,365	37,673	37,438
37,63	37,24	37,387	38,071	37,419	37,403	37,73	37,317
37,572	37,409	37,387	38,078	37,587	37,39	37,806	37,457
37,592	37,616	37,303	38,44	37,133	37,195	37,637	37,558
37,216	37,447	37,361	38,522	37,313	37,176	37,618	37,221
37,261	37,478	37,412	38,49	37,475	37,296	37,719	37,379
37,471	37,553	37,425	38,471	37,721	37,7	37,687	37,493
37,382	37,434	37,444	38,408	38,131	37,624	37,567	37,607
37,445	37,622	37,432	37,328	38,225	37,75	37,808	37,69
37,382	37,447	37,47	37,341	37,995	37,782	37,89	37,798
37,382	37,29	37,586	37,24	38,032	37,769	37,852	37,779
37,566	37,422	37,798	37,741	37,765	37,712	37,94	37,867
37,382	37,578	37,81	37,894	37,721	37,542	37,877	37,766
37,458	37,603	37,727	37,938	37,982	37,668	38,079	38,026
37,458	37,478	37,695	38,071	38,026	37,977	37,839	37,626
37,261	37,478	37,907	38,021	38,15	38,287	37,694	38,045
37,547	37,321	37,695	38,129	38,057	38,287	37,637	38,045
37,884	37,491	38,221	38,122	38,293	38,432	37,808	37,988
37,878	37,622	38,17	37,938	38,368	38,463	37,852	37,912
37,897	37,76	38,074	38,192	38,343	38,47	37,972	37,874
37,522	37,628	37,855	38,046	37,945	38,545	38,067	37,626

37,693	37,597	37,855	38,002	38,38	38,331	38,004	38,07
38,005	37,422	37,984	38,084	37,833	38,249	38,345	38,196
37,77	37,716	38,234	38,224	38,044	38,419	38,332	38,145
37,954	37,478	38,407	38,224	38,108	38,533	38,294	38,031
37,838	37,491	38,414	38,287	38,139	38,463	38,231	38,107
37,552	37,578	38,407	38,236	38,12	38,451	38,079	38,056
37,265	38,054	38,35	38,205	37,928	38,419	38,269	38,082
37,609	37,81	38,234	38,103	38,201	38,57	38,427	38,196
37,539	37,653	38,266	38,275	38,282	38,47	38,554	38,475
37,526	37,954	38,234	38,529	38,164	38,451	38,604	38,437
37,475	37,754	38,144	38,275	38,002	38,406	38,516	38,272
37,539	37,553	38,17	38,129	38,008	38,577	38,32	38,126
37,564	37,459	38,061	38,217	37,934	38,331	37,991	37,923
37,628	37,585	37,663	38,211	37,803	37,946	37,795	37,79
37,488	37,772	37,412	38,065	37,754	38,059	37,472	37,581
37,552	37,653	37,624	37,906	37,648	37,977	37,238	37,346
37,443	37,553	37,522	37,519	37,511	37,977	37,339	37,403
37,361	37,422	37,207	37,405	37,287	37,82	37,264	37,39
37,297	37,346	37,265	37,271	37,126	37,7	37,289	37,219
37,278	37,221	37,194	37,246	37,169	37,535	37,226	37,188
37,163	37,14	37,271	37,112	37,157	37,296	37,156	37,2
37,093	37,215	37,181	37,1	37,063	37,144	37,162	36,934
37,081	37,215	37,188	36,96	36,902	37,112	37,131	36,629
36,851	37,158	36,905	36,941	36,777	37,087	37,055	36,655
36,794	36,789	36,758	36,865	36,721	37,1	36,985	36,782
36,839	36,901	36,803	36,878	36,752	37,112	36,935	36,744
36,826	37,021	36,912	37,068	36,79	37,112	36,96	36,813
36,794	37,127	36,95	37,125	36,796	37,024	36,973	36,839
36,858	37,19	37,002	37,1	36,827	36,809	36,998	36,807
36,832	37,252	36,989	36,827	36,852	36,763	37,004	36,837
36,603	37,265	37,059	36,801	36,653	36,567	36,776	36,825
36,73	37,315	37,034	36,598	36,54	36,535	36,675	36,647
36,788	36,77	37,034	36,395	36,571	36,807	36,565	36,527
36,832	36,563	37,034	36,522	36,596	36,939	36,438	36,552
36,953	36,736	36,989	36,646	36,621	36,952	36,457	36,596
37,061	36,824	36,989	36,684	36,695	36,971	36,362	36,634
37,125	36,906	37,002	36,703	36,683	36,984	36,375	36,679
36,953	37,031	37,034	36,869	36,826	36,971	36,407	36,71
36,82	36,83	37,014	36,881	36,764	36,889	36,539	36,647
36,807	36,912	36,925	36,926	36,639	36,807	36,495	36,469
36,928	36,924	36,963	36,996	36,82	36,775	36,577	36,596
36,775	36,968	36,88	37,053	36,82	36,838	36,647	36,603
36,737	36,937	36,828	37,078	36,82	37,009	36,761	36,647
36,673	36,799	36,815	36,996	36,956	37,034	36,805	36,755
36,667	36,837	36,732	37,123	37,043	37,034	36,982	36,806
36,641	36,893	36,687	37,148	37,112	37,053	36,932	36,888
36,718	36,962	36,674	36,716	37,162	37,116	36,97	37,009
36,826	37,019	36,7	36,742	37,162	36,952	37,065	37,002
37,023	37,075	36,661	36,976	37,205	36,826	37,242	36,99
36,985	37,075	36,661	36,945	36,913	36,801	36,957	36,996
37,068	37,031	36,745	37,015	36,788	36,801	36,906	37,028
37,125	37,087	36,7	36,716	36,757	36,952	36,862	36,977
37,125	37,075	36,745	36,913	36,9	36,952	36,932	37,104
37,221	37,075	36,822	36,684	36,788	36,996	36,995	36,825
37,17	37,087	36,854	36,684	36,795	37,047	37,001	36,913
37,17	37,062	36,835	36,672	36,838	37,053	37,09	36,977
36,941	37,087	37,014	36,691	36,863	37,173	37,166	37,002
36,998	37,031	36,867	36,716	36,925	37,11	37,052	37,104
37,081	37,031	37,014	36,799	36,956	36,952	37,096	37,218
36,82	36,874	37,014	36,716	36,967	36,756	36,995	37,218
36,656	36,661	36,88	36,703	37,042	36,693	36,812	37,173
36,548	36,674	36,925	36,849	37,092	36,763	36,805	37,085
36,471	36,724	36,925	36,914	37,061	36,441	36,875	37,135
36,388	36,661	36,764	36,864	37,104	36,567	36,944	36,952
36,439	36,661	36,604	36,876	36,831	36,592	36,97	36,825
36,548	36,711	36,539	36,889	36,725	36,649	36,957	36,73
36,592	36,774	36,604	36,844	36,7	36,58	37,039	36,685

36,669	36,906	36,604	36,756	36,7	36,731	37,096	36,71
36,707	36,818	36,578	36,717	36,713	37,116	36,982	36,793
36,77	36,724	36,648	36,73	36,762	37,344	37,001	36,837

Fig S1									
Mean Temp, control group	SE	Mean Temp, L-17 140 mg/kg	SE	140 mg/kg L-17 Mouse#1 T °C	140 mg/kg L-17 Mouse#2 T °C	140 mg/kg L-17 Mouse#3 T °C	Control Mouse#4 T °C	Control Mouse#5 T °C	Control Mouse#6 T °C
35,82167	0,048704	37,15967	0,193646	36,446	36,973	37,312	35,853	37,308	36,209
35,90867	0,021528	37,08567	0,206042	36,874	37,274	37,326	35,885	37,168	36,29
35,88600	0,030512	37,08333	0,121743	36,867	37,001	37,258	35,972	37,382	36,265
35,85700	0,074782	36,62933	0,152600	36,819	36,966	37,157	35,785	37,431	36,876
35,76933	0,107465	36,52400	0,319535	36,84	36,764	37,083	35,592	37,449	37,672
36,15433	0,002963	36,41433	0,387719	37,171	36,1	37,016	35,598	37,357	37,772
36,27467	0,144861	36,19100	0,414826	37,054	36,505	37,211	35,685	36,875	37,89
36,45867	0,193978	36,15333	0,433501	37,04	36,533	37,326	35,991	37,73	38,046
36,48300	0,288718	35,98800	0,396890	36,619	37,407	37,434	36,813	38,017	38,108
36,73633	0,248458	35,99033	0,386175	36,647	37,644	37,515	36,744	37,962	38,077
37,04633	0,142193	35,73433	0,387192	37,012	36,959	37,434	37,186	37,791	37,934
37,10300	0,324116	35,93300	0,398586	37,123	36,456	36,975	36,788	37,412	37,703
37,28500	0,354514	36,09300	0,480032	36,819	36,17	36,813	37,043	37,052	37,504
37,15200	0,450755	36,56867	0,450064	36,516	36,198	36,624	36,894	37,003	37,255
37,09400	0,462496	37,15400	0,390649	36,447	36,267	36,449	36,838	37,156	36,938
37,07333	0,459169	37,49200	0,376453	36,178	36,309	36,496	36,744	37,608	36,682
37,43667	0,438371	37,65067	0,302946	35,937	35,981	36,341	36,757	37,822	36,533
37,29833	0,320182	37,82933	0,270817	35,909	36,12	36,449	36,682	37,486	36,471
37,41567	0,293603	38,00233	0,332879	35,874	35,974	36,402	36,364	37,418	36,303
37,33567	0,117133	37,81600	0,276156	35,771	35,953	36,267	36,078	37,046	36,165
37,58800	0,378028	37,05300	0,217263	35,674	35,876	36,334	35,617	37,431	35,898
38,00867	0,354050	36,07200	0,327907	35,509	35,799	36,429	35,592	37,638	35,779
38,43900	0,397123	35,38633	0,292090	35,454	35,897	36,415	35,536	37,724	35,898
38,41267	0,339550	34,50767	0,436487	35,44	35,771	36,28	35,236	37,431	36,128
38,18767	0,355278	33,44367	0,565764	35,261	35,862	36,226	35,336	37,412	36,134
38,08333	0,471650	32,45267	0,482082	35,178	35,925	36,247	35,292	37,278	35,972
37,96967	0,372597	31,97467	0,574623	35,219	36,051	36,105	35,299	37,4	35,941
38,01600	0,264593	31,53833	0,639485	35,281	35,988	36,132	35,43	37,247	35,792
37,76300	0,218500	31,13467	0,697587	35,447	36,058	36,199	35,274	37,345	35,692
37,71700	0,251162	30,78600	0,746058	35,523	36,148	36,152	35,554	37,156	35,742
37,39200	0,135000	30,47400	0,780203	35,73	35,988	36,645	35,536	37,272	35,829
37,42400	0,300200	30,20367	0,805438	35,799	35,925	36,793	35,81	37,321	35,792
37,59800	0,546731	30,00433	0,824044	35,722	35,813	36,786	35,966	37,071	35,929
37,62033	0,265889	29,85800	0,830717	35,66	35,771	36,867	36,097	36,655	35,935
37,62800	0,348675	29,73300	0,807763	35,619	35,911	36,685	36,24	36,563	35,997
37,39600	0,293159	29,78300	0,786914	35,584	36,169	36,55	35,779	36,27	36,284
37,26067	0,187839	29,84633	0,734170	35,419	36,274	36,489	35,885	35,916	36,795
37,25733	0,185050	29,91367	0,676502	35,667	36,092	36,726	35,966	35,83	37,324
37,22467	0,110140	30,06767	0,572809	35,736	35,925	37,09	36,713	35,867	37,753
37,13533	0,070219	30,13033	0,458185	36,198	36,386	37,285	36,906	36,906	37,946
37,06633	0,053797	30,48700	0,332425	36,612	36,728	37,36	36,676	37,633	37,828
36,95833	0,099593	30,96233	0,238617	36,77	36,861	37,461	37,136	37,779	37,697

37,08067	0,101952	31,50500	0,243056	36,784	36,968	37,427	37,068	37,749	37,697
37,16867	0,142368	32,01767	0,422381	36,722	36,667	37,535	36,788	37,675	37,56
37,46133	0,255357	32,59467	0,436667	36,577	37,072	37,616	36,632	37,761	37,473
37,66900	0,332650	33,12967	0,529486	36,115	36,709	37,434	36,769	37,675	37,405
37,92933	0,237313	33,83467	0,688432	35,729	36,234	37,373	37,112	37,511	37,417
38,00733	0,309406	34,31300	0,616631	35,439	36,052	37,4	37,136	37,058	37,535
37,49467	0,190639	34,87367	0,512670	35,329	35,976	37,137	36,601	36,606	37,237
37,22767	0,112014	35,27733	0,376234	35,412	35,878	36,665	37,093	36,252	36,857
36,99100	0,145139	35,38767	0,244652	35,481	36,094	36,422	36,744	36,13	36,851
36,93767	0,143947	35,44167	0,109651	35,57	35,997	36,112	36,358	35,934	36,863
37,01400	0,120355	35,61167	0,222684	35,639	35,892	36,091	36,171	35,873	36,626
36,82367	0,080429	35,90600	0,403163	35,701	35,696	35,916	36,009	35,787	36,502
36,91433	0,215781	35,93767	0,273332	35,757	35,654	35,909	35,891	35,567	36,334
37,09767	0,230088	36,11467	0,233899	35,674	35,829	35,936	35,741	35,5	36,097
37,09900	0,283810	36,09400	0,227386	35,577	35,745	36,004	35,766	35,347	35,835
37,35133	0,314691	36,29033	0,518137	35,564	35,724	35,97	35,554	35,463	35,736
37,52367	0,402758	36,37167	0,580275	35,398	35,731	36,159	35,33	35,604	35,586
37,28833	0,243971	36,24000	0,273615	35,398	35,668	36,186	35,529	35,616	35,411
37,29900	0,368587	36,46967	0,278478	35,301	35,906	35,984	35,567	35,543	35,443
37,09333	0,169400	36,69867	0,245508	35,267	36,15	35,896	35,554	35,555	35,655
37,22300	0,224812	36,86367	0,376479	35,343	36,052	35,822	35,629	35,445	35,611
37,80000	0,348861	36,82167	0,348403	35,329	35,913	35,943	35,642	35,365	35,673
37,55567	0,215517	36,82933	0,338834	35,198	35,892	35,828	35,598	35,378	35,673
37,35033	0,181760	36,83767	0,276486	35,218	35,626	35,795	35,716	35,402	35,673
37,27567	0,217236	37,11800	0,335831	35,149	35,473	35,761	35,604	35,592	35,63
37,67167	0,272813	37,38867	0,525770	34,901	35,487	35,808	35,536	35,818	35,574
37,57400	0,324288	37,23967	0,497544	34,818	35,466	35,835	35,598	35,909	35,561
37,56500	0,527876	37,23833	0,513304	35,087	35,27	35,801	35,835	35,94	35,686
37,55433	0,206980	37,23567	0,455966	35,349	35,141	35,72	35,941	36,276	35,729
37,55467	0,116700	37,11033	0,393264	35,349	34,994	35,768	36,307	37,058	36,401
37,48467	0,319005	37,20300	0,386002	35,432	34,952	35,727	36,55	37,59	36,98
37,48167	0,377100	37,70433	0,433368	35,349	35,029	36,004	36,749	37,602	37,185
37,45133	0,343120	37,74800	0,374349	35,646	35,225	35,822	36,631	37,407	37,31
37,67533	0,374195	37,66267	0,332461	35,356	35,162	36,152	35,983	36,906	37,36
37,98400	0,425129	37,54333	0,379955	35,356	35,392	36,152	36,182	36,887	37,304
37,66067	0,201598	37,57767	0,454577	35,591	35,728	36,078	36,687	37,242	37,434
37,59633	0,235900	37,57200	0,441168	35,584	35,707	36,064	36,817	37,352	37,347
37,77867	0,169751	37,79600	0,439456	35,446	35,637	35,984	36,662	37,401	37,279
37,87767	0,314965	37,90033	0,437716	35,501	35,539	35,754	36,388	37,205	36,936
37,91267	0,199026	38,14100	0,363443	35,467	35,364	35,613	36,375	37,04	36,631
37,80833	0,176765	38,01233	0,267323	35,46	35,462	35,727	36,338	36,82	36,314
37,90600	0,221276	38,07067	0,398229	35,425	35,574	35,673	36,276	36,655	36,12
38,26600	0,399945	38,01467	0,330417	35,184	35,574	35,849	35,914	36,46	36,133
38,13167	0,302528	37,90900	0,310511	35,156	35,553	35,923	35,59	36,313	36,251
37,92433	0,134923	37,89367	0,374443	35,218	35,567	35,876	35,92	36,233	36,413

38,13767	0,224500	37,92633	0,432192	35,377	35,965	35,923	35,621	35,982	36,451
38,24033	0,219837	37,81633	0,396625	35,218	36,217	36,044	35,534	35,848	36,282
37,75033	0,409801	37,88233	0,439336	35,136	36,098	36,132	35,303	35,781	36,145
37,76533	0,166054	37,81733	0,307509	35,239	35,951	36,044	35,154	35,781	36,077
37,70800	0,374992	37,99433	0,369529	35,356	36,035	35,795	35,603	35,805	35,884
37,72367	0,252595	38,22033	0,309628	35,529	35,902	36,489	35,74	35,744	35,803
37,72633	0,090223	38,36033	0,319634	35,784	35,951	36,773	35,609	35,805	35,74
37,84000	0,413384	38,18500	0,484674	36,184	36,182	36,8	35,528	35,707	35,709
37,94133	0,193834	38,17700	0,602131	36,301	36,238	36,793	35,484	35,677	35,79
38,05733	0,390360	37,98400	0,483704	36,246	36,126	36,604	35,896	35,622	35,747
38,14100	0,301885	38,14000	0,462083	36,073	36,161	36,476	35,503	35,664	35,622
37,99567	0,294586	38,22333	0,361479	35,846	36,099	36,334	35,472	35,854	35,666
				36,17	36,491	36,739	35,54	35,903	35,678
				36,487	36,826	37,211	35,54	35,885	35,691
				36,598	37,07	37,366	35,571	36,056	35,79
				36,653	37,161	37,339	35,497	35,878	35,784
				36,763	37,161	37,427	35,553	36,019	36,002
				36,756	37,357	37,427	35,447	36,288	36,027
				36,784	37,594	37,71	35,403	36,367	36,089
				36,846	37,713	37,656	35,447	36,3	35,958
				36,935	37,322	37,623	35,247	36,337	36,114
				36,86	37,525	37,582	35,44	36,251	36,519
				36,508	37,511	37,805	35,571	36,19	36,855
				36,273	37,35	37,454	35,378	36,429	37,154
				35,963	36,623	37,353	35,578	37,095	37,31
				35,853	36,463	37,373	35,665	37,425	36,968
				35,867	36,344	37,178	36,082	37,345	36,818
				35,929	36,274	36,651	36,736	36,862	36,476
				35,825	36,051	36,395	36,338	36,477	36,22
				35,839	36,281	36,361	36,369	36,27	36,395
				36,06	36,924	36,341	36,263	36,374	36,507
				36,149	36,623	36,159	36,132	36,41	36,613
				36,536	36,337	36,139	36,413	36,508	36,936
				36,805	36,623	36,28	36,562	36,569	37,341
				36,894	36,742	36,456	36,406	37,046	37,478
				37,411	37,077	37,063	36,824	37,125	37,055
				38,163	37,594	38	36,35	36,875	36,924
				38,301	37,664	38,526	36,369	38,182	37,459
				38,225	37,916	38,52	37,122	38,756	38,119
				38,039	37,646	38,702	37,291	38,89	38,474
				38,129	37,814	38,446	37,199	38,915	38,551
				38,039	37,549	38,479	37,149	39	38,588
				38,177	37,514	38,634	37,143	39,183	38,427
				38,273	37,493	38,688	37,112	39,079	38,458
				38,294	37,598	38,338	37,211	39,055	38,209

				38,205	37,416	38,338	37,124	39,018	38,296
				38,053	37,43	38,392	37,13	39,336	38,277
				37,97	37,325	38,257	36,838	39,183	38,34
				37,977	37,297	38,358	37,161	39,134	38,327
				37,818	37,611	38,243	37,329	38,896	38,476
				37,618	37,143	37,967	36,881	39,024	38,371
				37,253	37,248	38,075	36,62	39,262	38,277
				37,308	37,011	37,913	36,894	39,226	38,464
				37,508	37,451	38,048	37,161	39,165	38,526
				37,791	37,681	37,906	37,149	39,128	38,458
				37,584	37,15	37,967	37,068	38,939	38,103
				37,198	37,437	37,636	36,825	38,891	38,147
				37,15	37,996	37,744	37,205	38,921	38,321
				36,999	37,29	37,677	36,887	38,768	38,122
				36,675	37,737	37,441	37,08	38,366	38,147
				36,647	37,528	37,589	37,242	38,213	38,184
				36,55	37,381	37,616	37,112	38,091	38,122
				37,019	37,367	37,65	36,694	38,292	38,165
				37,275	37,632	37,495	36,676	38,243	38,016
				37,433	37,451	37,164	37,105	37,724	38,053
				37,585	36,773	37,184	37,497	37,364	37,686
				37,475	37,143	37,434	37,143	37,425	37,263
				37,681	36,869	37,501	35,972	36,869	37,294
				37,799	37,002	37,832	35,659	36,796	37,019
				37,764	36,974	37,393	35,609	36,728	36,956
				37,543	36,806	37,178	36,332	36,942	37,013
				37,681	37,107	37,11	36,811	37,895	36,994
				37,316	38,029	36,8	36,911	37,859	36,944
				36,833	37,847	37,454	36,163	38,225	37,093
				36,35	37,421	37,454	36,288	38,243	37,809
				36,047	37,344	37,481	36,4	37,529	38,232
				36,157	37,281	37,373	36,506	37,333	38,189
				36,647	36,911	37,501	36,301	37,492	38,195
				36,937	37,575	37,67	36,363	38,952	38,332
				37,24	37,575	37,616	36,494	39,007	38,394
				37,054	36,792	37,69	37,073	38,701	38,487
				37,15	36,68	37,542	37,135	38,933	38,705
				37,419	37,002	37,69	37,44	38,738	38,537
				37,606	37,33	37,919	37,521	38,83	38,599
				37,633	37,379	38,129	37,552	38,848	38,662
				37,868	37,239	38,25	36,967	38,823	38,568
				37,999	37,561	38,277	37,16	38,854	38,55
				38,075	37,728	38,041	37,427	38,878	38,519
				37,951	37,833	38,385	37,197	38,768	38,506
				38,041	37,889	37,994	37,16	38,231	38,463

				37,868	37,735	38,243	37,154	38,103	38,525
				38,013	37,861	38,176	37,452	38,274	38,531
				37,924	37,756	38,27	37,608	38,451	38,531
				37,82	37,547	38,182	37,838	38,5	38,543
				38,137	37,617	38,156	37,396	38,402	38,363
				37,999	37,303	38,169	37,203	38,5	38,344
				37,951	37,442	38,081	37,454	38,274	38,539
				38,082	37,701	38,102	37,56	38,164	38,389
				38,075	37,764	38,081	37,236	37,767	38,24
				38,137	37,701	38,216	37,578	37,468	38,34
				37,972	37,792	38,102	37,491	37,511	38,327
				37,924	37,715	38,135	37,448	37,816	38,47
				37,986	37,687	38,095	37,13	38,176	38,458
				38,103	37,554	38,081	37,354	38,28	38,501
				37,882	37,568	38,041	36,782	38,28	38,277
				37,703	37,477	37,973	37,174	38,341	38,489
				37,634	37,47	38,095	37,143	37,932	38,427
				37,634	37,638	38,108	37,149	38,256	38,495
				37,661	37,659	38,108	37,074	38,17	38,209
				37,868	38,057	38,122	36,707	38,146	38,302
				37,813	37,393	38,081	36,993	37,999	38,165
				37,793	37,561	37,933	37,012	38,139	38,14
				37,806	37,743	37,798	36,8	38,579	38,078
				37,626	37,708	37,724	36,807	38,933	38,172
				37,626	37,715	38,075	37,404	38,548	38,184
				37,426	37,624	38,088	37,105	38,347	38,172
				37,571	37,177	37,764	37,031	38,115	38,19
				37,488	37,079	37,71	36,912	38,231	38,315
				37,737	37,135	37,778	36,881	38,249	38,196
				37,585	37,184	37,953	36,782	38,133	38,346
				37,875	37,128	37,98	36,925	38,212	38,215
				38,061	37,33	37,919	36,906	38,219	38,265
				38,006	37,407	38,088	37,08	38,341	38,321
				37,751	37,022	38,027	37,51	38,109	38,389
				37,84	37,588	37,528	37,28	38,078	38,315
				37,833	37,246	38,061	37,112	38,127	38,383
				37,557	37,344	38,048	36,813	38,066	38,302
				37,668	36,959	37,879	36,439	38,2	38,147
				37,895	37,288	37,548	36,396	37,938	38,153
				37,819	38,259	37,434	36,402	37,773	38,147
				38,054	37,351	37,515	36,57	38,621	38,178
				37,95	37,183	37,515	36,744	38,493	38,14
				38,04	37,113	37,481	37,006	38,707	38,14
				37,916	37,428	37,387	37,205	38,554	38,209
				38,061	37,372	37,4	37,261	38,676	38,203

				37,813	37,511	37,521	37,105	38,658	38,371
				37,751	37,462	37,555	37,529	38,86	38,464
				37,44	37,588	37,582	37,466	38,304	38,228
				37,275	37,574	37,609	37,249	38,615	38,234
				37,219	37,099	37,656	37,385	38,634	38,308
				37,295	37,686	37,879	37,479	38,42	38,228
				37,212	37,539	37,811	37,354	38,505	38,246
				36,992	37,064	37,791	36,919	38,597	38,271
				36,785	37,428	37,953	37,529	38,591	38,321
				36,537	36,841	37,683	37,392	38,585	38,377
				36,495	36,994	38,135	37,28	38,683	38,333
				36,219	37,518	37,98	37,491	38,927	38,315
				36,047	37,749	38,061	37,311	38,786	38,327
				36,054	37,253	38,068	37,261	38,646	38,29
				36,081	37,141	37,832	37,043	38,683	38,165
				35,943	36,694	37,872	37,099	38,597	38,109
				35,716	36,378	37,906	37,112	38,573	38,14
				35,943	36,378	37,704	37,149	38,658	38,022
				36,109	36,267	37,488	37,149	38,713	38,078
				36,137	36,413	37,67	37,13	38,512	38,147
				36,288	36,476	37,798	37,211	38,505	38,091
				36,274	36,511	37,737	37,286	38,463	38,24
				36,109	36,658	37,697	37,186	38,133	38,221
				35,957	36,874	37,67	37,23	38,255	38,34
				36,026	36,909	37,758	37,193	38,322	38,364
				36,261	36,679	37,825	37,385	38,469	38,514
				36,688	36,462	37,967	37,186	38,518	38,203
				37,219	36,26	37,967	37,024	38,567	38,153
				37,405	36,323	37,778	37,124	38,481	37,904
				37,626	36,525	37,596	37,018	38,322	37,724
				37,75	36,833	37,778	36,707	38,102	37,462
				37,543	37,587	37,596	36,396	37,907	37,238
				37,557	36,86	37,785	36,252	37,669	36,877
				37,481	36,993	37,744	36,103	37,706	36,696
				37,537	37,063	37,778	35,966	38,036	36,528
				37,13	37,035	37,629	36,009	38,207	36,441
				36,765	36,693	37,4	36,196	38,024	36,341
				36,496	36,644	37,38	35,978	37,724	36,279
				36,351	37,014	37,589	36,103	37,755	36,291
				36,206	36,595	37,488	36,234	37,614	36,329
				36,02	36,476	37,562	36,022	37,449	36,192
				36,047	36,365	37,481	35,991	37,248	36,086
				36,123	36,455	37,677	36,047	37,315	36,005
				36,13	36,392	37,724	36,04	37,37	36,173
				36,144	36,323	37,501	36,283	37,401	36,111

				36,103	36,24	37,387	35,916	37,095	35,923
				36,123	36,498	37,09	35,835	37,034	35,91
				36,068	36,428	36,726	36,221	36,655	35,929
				35,999	36,386	36,631	36,196	36,233	36,028
				35,958	36,212	36,476	36,153	36,233	35,979
				35,903	36,205	36,395	36,065	36,148	35,91
				35,992	36,24	36,314	35,978	36,093	36,016
				36,054	36,33	36,172	36,128	36,099	36,097
				36,351	36,079	36,246	36,333	35,946	36,122
				36,427	35,988	36,563	36,352	35,952	36,141
				36,144	36,079	36,516	35,816	36,081	36,452
				35,93	36,254	36,368	36,159	36,166	36,956
				35,847	36,261	36,145	36,358	36,288	37,38
				35,785	36,365	36,044	36,676	36,661	37,05
				35,806	36,666	36,199	36,694	36,466	36,913
				35,827	36,659	36,24	36,377	36,264	36,651
				35,758	36,798	36,887	36,315	36,27	36,545
				36,205	36,666	37,332	36,315	36,056	36,527
				36,757	37,001	37,508	36,234	36,252	36,433
				37,074	37,183	37,528	36,153	36,435	36,327
				37,495	37,211	37,541	35,829	36,337	36,446
				37,591	37,399	37,656	35,928	36,27	36,583
				37,584	37,357	37,906	36,097	36,117	37,149
				37,516	37,106	37,69	36,016	36,038	37,635
				37,467	37,183	37,764	35,754	35,879	37,915
				37,488	37,553	37,757	36,202	36,838	38,058
				37,543	37,001	37,737	36,433	37,743	37,89
				37,364	36,764	37,643	36,663	37,816	37,909
				37,433	36,876	37,501	36,52	37,871	37,91
				37,198	36,764	37,211	36,701	37,81	37,985
				36,929	36,995	37,622	36,95	37,834	37,96
				36,612	37,162	37,258	36,813	37,834	37,867
				36,488	36,946	37,299	36,8	37,456	37,742
				36,212	36,897	37,218	36,707	37,431	37,543
				36,171	36,68	37,036	36,47	37,15	37,089
				36,026	36,576	36,935	35,966	36,814	36,784
				35,826	36,492	36,894	35,959	36,295	36,572
				35,729	36,324	36,651	35,991	35,977	36,509
				35,674	36,177	36,591	35,91	36,172	36,229
				35,722	36,094	36,543	35,941	36,105	36,067
				35,812	36,045	36,361	35,978	36,013	36,024
				35,688	35,926	36,328	35,991	36,001	35,943
				35,729	35,898	36,26	35,673	36,081	36,061
				35,626	35,947	36,098	35,847	36,147	36,086
				35,571	36,003	36,085	35,953	35,995	36,061

				35,432	36,205	36,368	35,878	36,001	35,999
				35,549	36,157	36,159	35,991	35,866	36,042
				35,418	36,219	36,193	36,065	35,872	36,154
				35,418	36,24	36,193	36,059	35,829	36,366
				35,487	36,296	36,058	36,121	35,799	36,161
				35,487	36,289	36,253	35,853	35,854	36,08
				35,667	36,275	36,456	35,573	35,848	36,235
				35,77	36,233	36,584	36,402	35,872	36,316
				35,729	36,073	36,28	36,738	35,762	36,248
				35,667	35,94	36,456	37,105	36,924	36,653
				35,97	35,945	36,746	37,006	37,669	37,58
				36,501	36,637	37,103	36,838	37,877	38,122
				36,701	36,658	37,339	36,744	37,907	38,116
				37,122	36,783	37,366	36,987	37,925	38,109
				37,067	36,727	37,103	36,925	37,834	38,035
				36,591	36,266	37,326	36,919	37,602	38,072
				36,584	36,162	37,029	36,825	37,669	37,842
				36,784	35,875	36,766	36,632	37,137	37,556
				36,667	35,735	36,395	36,308	37,455	37,313
				36,508	36,029	36,274	36,302	37,272	37,07
				36,577	35,98	36,159	36,184	37,162	36,833
				36,673	35,98	36,287	36,333	36,71	36,703
				36,715	35,889	36,118	36,271	36,655	36,416
				36,646	35,98	36,22	35,959	37,143	36,148
				36,804	36,238	36,038	36,009	37,504	36,024
				36,425	35,889	36,341	35,966	37,382	36,055
				36,308	35,805	36,368	35,704	37,131	35,862
				36,122	35,847	36,415	35,635	37,034	36,03
				35,784	35,847	36,442	35,666	36,777	35,943
				35,549	35,763	36,429	35,567	36,471	35,581
				35,529	35,687	36,449	35,523	36,251	35,419
				35,494	35,693	36,382	35,635	35,94	35,519
				35,356	35,659	36,355	35,723	35,817	35,643
				35,308	35,624	36,334	35,704	35,842	35,612
				35,287	35,596	36,355	35,791	35,866	35,525
				35,425	35,617	36,321	35,872	35,946	35,587
				35,384	35,568	36,132	35,966	36,068	35,619
				35,377	35,924	35,977	35,947	36,178	35,668
				35,439	35,911	36,145	35,598	36,215	35,394
				35,563	36,037	36,314	35,677	35,964	35,317
				35,474	36,044	36,199	35,733	35,756	35,267
				35,474	36,085	36,118	35,877	35,811	35,304
				35,432	35,946	36,145	35,752	35,658	35,435
				35,225	35,806	36,159	35,758	35,603	35,641
				35,253	35,792	36,139	35,983	35,56	35,983

				35,246	35,925	35,788	35,702	35,444	36,251
				35,467	35,995	36,011	35,528	35,762	36,17
				35,418	35,806	36,118	35,465	35,854	36,139
				35,453	35,715	36,058	35,353	35,97	35,99
				35,46	35,897	35,957	35,534	36,16	35,74
				35,481	35,925	35,721	35,74	36,85	35,697
				35,549	35,974	35,606	35,721	36,685	35,747
				35,46	35,897	36,038	35,864	36,618	35,722
				35,384	36,009	36,55	35,559	36,484	35,809
				35,184	36,085	36,833	35,515	36,258	35,803
				35,17	36,26	37,13	35,565	36,105	36,039
				35,377	36,078	37,285	35,44	35,971	35,846
				35,563	35,96	37,103	35,434	35,861	35,834
				35,674	36,281	36,813	35,503	35,854	35,934
				35,674	36,246	36,618	35,447	35,83	36,133
				35,715	36,519	36,618	35,235	35,494	36,064
				35,736	36,176	36,51	35,253	35,543	36,033
				35,818	36,4	36,213	35,204	35,549	35,99
				35,432	36,295	36,132	35,341	35,555	35,977
				35,377	36,058	36,179	35,565	35,585	36,114
				35,336	36,085	36,166	35,54	35,689	36,033
				35,515	36,408	36,105	35,241	35,799	35,778
				35,439	36,52	36,051	35,366	35,891	36,091
				35,763	36,233	35,984	35,403	35,946	36,016
				35,722	35,968	36,017	35,434	36,099	35,792
				35,598	35,968	36,22	35,334	35,946	36,016
				35,384	35,884	36,152	35,403	35,75	36,141
				35,563	35,954	36,112	35,328	35,573	36,159
				35,749	35,856	36,213	35,403	35,469	36,153
				35,825	36,087	36,159	35,515	35,494	36,153
				35,66	36,212	36,395	35,528	35,598	36,078
				35,687	36,177	36,294	35,185	35,677	36,078
				35,784	36,031	36,483	35,023	35,781	36,159
				36,205	36,499	36,476	35,222	35,891	36,165
				36,549	36,646	37,11	35,347	35,695	36,159
				36,839	36,715	37,326	35,384	35,714	36,035
				36,86	36,513	37,015	35,565	35,909	35,979
				36,37	36,457	36,557	35,927	35,774	36,228
				35,88	36,219	36,786	36,444	36,215	36,726
				35,749	36,548	37,278	36,849	37,217	37,224
				35,867	36,82	37,231	37,129	37,657	37,641
				36,349	36,715	37,252	37,154	37,406	37,784
				36,474	36,666	36,948	37,147	37,455	37,915
				36,377	36,736	36,779	37,166	37,394	37,772
				36,225	36,639	36,705	37,141	37,07	37,741

				36,018	36,506	36,665	36,587	36,82	37,766
				35,936	36,694	36,611	36,419	36,539	37,629
				35,798	36,848	36,537	36,307	36,49	37,629
				36,225	37,002	37,616	35,827	36,41	37,318
				36,522	37,178	37,906	36,251	36,667	36,925
				36,825	37,261	37,933	36,701	37,266	37,375
				37,108	37,373	37,912	36,676	37,461	37,674
				37,349	37,513	38,283	36,85	37,944	37,73
				37,301	37,583	38,189	36,8	37,388	37,556
				37,377	37,534	38,243	36,819	37,07	37,319
				37,356	37,52	38,034	36,794	37,04	37,3
				37,336	37,799	38,014	37,074	37,027	37,163
				37,177	37,331	37,852	37,205	37,357	36,995
				37,115	37,45	37,447	37,168	37,705	36,958
				37,094	36,982	37,238	36,744	37,877	37,25
				36,908	36,758	36,861	36,956	38,164	37,58
				36,936	36,549	36,584	36,844	37,834	37,748
				36,894	36,416	36,564	36,863	37,351	37,724
				36,584	36,689	36,591	36,726	36,704	37,786
				36,26	36,975	36,746	36,427	36,728	37,761
				36,508	37,171	37,427	36,526	37,302	37,73
				36,846	37,506	37,798	36,794	37,486	37,705
				37,019	37,408	38,048	36,869	37,858	37,73
				37,467	37,597	38,156	37,08	37,272	37,885
				37,592	37,373	38,297	37,373	37,968	37,941
				37,619	37,632	38,25	37,448	37,657	37,985
				37,729	37,359	38,088	37,286	37,84	38,091
				37,536	37,352	37,899	37,516	37,962	38,072
				37,86	37,157	37,744	37,305	37,773	38,122
				37,612	37,366	38,108	37,267	37,437	38,035
				37,247	37,499	37,744	37,205	37,143	38,091
				36,84	37,178	37,899	37,161	37,443	38,128
				36,798	37,114	37,4	36,757	37,088	37,767
				36,564	37,526	37,097	37,074	36,948	37,661
				36,529	37,051	36,753	37,317	36,887	37,512
				36,895	37,323	36,415	37,348	37,082	37,145
				37,15	37,218	36,51	37,174	37,492	37,064
				37,543	36,967	36,705	37,105	37,809	37,313
				37,826	37,225	37,205	37,323	37,925	37,499
				38,005	36,946	37,555	37	37,797	37,618
				37,854	37,994	37,845	37,149	37,742	37,73
				38,109	37,617	38,075	37,161	37,687	37,692
				38,109	37,554	38,324	37,062	37,638	37,748
				38,129	37,463	38,223	37,224	37,559	37,661
				37,998	37,225	38,176	36,713	37,315	37,643

				37,764	37,414	38,182	36,732	37,308	37,543
				37,847	37,596	38,129	37,286	37,431	37,773
				37,888	37,148	37,919	36,894	37,577	37,717
				37,957	37,267	37,845	37,193	37,632	37,705
				37,771	37,309	37,778	37,236	37,864	37,736
				37,633	37,232	37,569	37,473	37,773	37,923
				37,792	37,232	37,697	37,093	38,096	37,624
				37,84	37,393	37,987	37,211	37,425	37,823
				38,054	37,623	37,926	37,541	37,602	37,711
				37,957	37,33	37,967	37,572	37,663	37,437
				37,978	37,519	38,027	37,522	37,877	37,705
				38,068	37,505	38,169	37,529	37,834	37,78
				38,116	37,344	38,014	37,08	37,534	37,916
				38,068	37,505	38,135	37,566	37,76	37,985
				38,047	37,575	38,203	37,423	37,803	37,773
				37,937	37,436	38,135	37,392	37,626	37,717
				37,826	37,827	37,906	37,155	37,718	37,711
				37,964	37,583	38,088	37,174	37,467	37,524
				38,019	37,562	37,96	37,273	37,125	37,655
				37,826	37,443	37,953	37,491	37,736	37,692
				37,923	37,862	37,913	37,217	37,73	37,593
				37,812	37,24	37,886	37,168	37,425	37,593
				37,709	37,548	37,987	37,118	37,565	37,356
				37,668	37,436	38,196	37,118	37,37	37,68
				37,799	37,464	37,906	37,087	37,412	37,537
				37,875	37,471	38,169	37,323	37,376	37,12
				37,909	37,848	38,061	37,136	37,596	37,506
				37,799	37,429	38,034	36,763	37,4	38,041
				37,964	37,974	37,785	36,838	37,62	37,748
				37,364	37,45	37,758	36,881	37,455	37,717
				37,04	37,136	37,609	37,205	37,363	37,705
				36,633	37,122	37,549	36,807	37,333	37,419
				36,309	37,143	37,852	37,006	37,401	37,319
				36,239	37,017	37,535	36,763	37,443	37,338
				36,315	37,108	37,4	36,887	37,339	37,63
				36,625	37,359	37,157	37,354	37,425	37,475
				36,501	37,017	37,582	37,317	37,028	37,537
				36,37	37,31	38,034	37,553	36,997	37,331
				36,549	37,324	37,839	37,13	37,474	37,96
				37,101	37,261	38,284	36,831	37,712	37,568
				37,405	37,213	38,311	36,576	38,17	37,35
				37,722	37,443	38,048	36,788	37,92	37,344
				37,977	37,499	37,69	36,564	37,914	37,288
				38,074	37,736	38,156	37,099	38,066	37,375
				37,97	37,52	37,946	37,23	37,81	37,568

				38,032	37,597	37,704	37,074	37,846	37,73
				37,984	37,338	37,832	37,51	37,926	37,356
				38,122	37,506	37,818	37,336	37,877	37,724
				38,129	37,569	37,845	37,516	37,846	37,556
				38,136	37,45	37,717	37,56	37,749	37,736
				37,825	37,715	38,183	37,323	37,804	37,724
				37,942	37,925	38,324	37,261	37,437	37,35
				37,811	37,632	38,102	37,317	37,718	37,4
				37,818	37,527	37,852	37,746	38,25	37,661
				38,129	37,799	38,196	37,703	37,889	37,655
				37,749	37,513	38,061	37,429	38,011	37,879
				37,763	38,163	37,96	37,417	38,231	37,425
				37,522	37,841	37,764	37,485	37,853	37,649
				37,439	37,289	37,414	37,236	37,651	37,674
				37,453	37,296	37,157	37,112	38,079	37,711
				37,474	37,478	37,13	37,012	37,529	37,661
				37,378	37,443	37,076	36,968	37,822	37,73
				36,854	37,394	37,009	37,392	37,682	37,941
				36,874	37,213	36,914	37,398	37,663	38,109
				36,743	37,366	37,076	37,255	37,749	37,966
				36,481	37,45	37,07	37,286	37,7	37,786
				36,136	37,674	36,975	37,149	37,883	37,836
				35,867	37,757	37,056	36,956	37,492	37,767
				35,426	37,639	37,434	37,373	37,761	38,084
				35,84	37,373	37,076	37,193	37,822	38,035
				36,088	36,863	36,914	37,454	37,767	37,966
				35,964	36,463	37,4	37,236	37,694	37,475
				35,874	36,197	37,312	37,463	37,449	37,636
				35,798	36,037	37,501	37,544	37,425	37,773
				35,702	36,03	37,798	37,5	37,333	37,792
				35,695	35,96	37,805	37,177	38,091	37,331
				35,888	35,967	37,798	37,146	37,346	36,977
				35,771	35,827	37,798	37,115	37,639	36,578
				36,033	35,96	37,872	37,233	37,443	36,485
				36,033	36,009	37,791	37,301	37,614	36,298
				35,964	36,134	37,865	37,115	37,376	36,229
				36,005	35,967	37,71	37,525	37,456	36,036
				36,04	36,477	37,852	36,778	37,058	36,098
				36,412	36,386	37,757	37,133	37,211	36,285
				37,026	37,056	37,609	37,183	37,456	36,522
				37,26	37,224	37,353	37,108	37,443	36,491
				37,64	37,294	37,36	37,096	37,425	36,927
				37,854	37,203	37,461	37,351	37,082	37,45
				37,798	37,427	37,447	36,81	36,899	37,742
				37,626	37,504	37,683	37,513	36,679	37,68

				37,599	37,336	37,515	37,146	36,691	37,985
				37,275	37,14	37,818	36,772	37,363	37,792
				37,247	37,029	37,528	36,909	37,381	37,686
				37,219	36,791	37,528	36,529	37,674	37,257
				36,95	37,154	37,407	36,311	37,729	36,914
				36,612	36,812	37,198	36,193	37,674	36,647
				36,399	36,861	37,11	35,994	37,467	36,709
				36,171	36,763	37,056	36	37,119	36,572
				35,978	36,7	37,076	36,075	37,302	36,497
				35,992	36,729	37,016	35,863	37,125	36,541
				36,192	36,862	36,786	36,064	37,4	36,564
				36,344	36,722	36,753	36,413	37,088	36,483
				36,213	36,436	36,766	36,456	37,699	36,309
				36,137	36,338	36,604	36,618	37,497	36,128
				36,088	36,247	36,847	36,587	37,418	36,053
				35,964	36,177	36,611	36,469	37,137	36,01
				35,654	36,261	36,516	36,444	37,009	35,879
				35,468	36,073	36,435	36,394	37,057	35,997
				35,295	35,968	36,334	36,251	36,783	35,785
				35,33	35,842	36,314	36,232	36,972	36,022
				35,302	35,626	36,955	35,553	36,618	35,972
				35,171	35,877	36,948	35,459	36,41	36,134
				35,495	35,842	36,914	35,958	36,385	36,165
				35,675	36,226	36,941	36,369	36,275	36,197
				35,702	36,597	36,773	36,525	36,098	36,29
				35,861	36,576	37,137	36,344	36,141	36,396
				35,902	36,52	37,002	36,313	36,532	36,165
				35,632	36,771	37,649	36,363	36,612	35,817
				35,522	36,932	38,122	36,662	36,649	35,717
				35,763	36,625	37,373	36,624	36,508	35,798
				36,067	36,436	37,063	36,687	36,202	35,891
				36,122	36,24	36,907	36,836	36,043	36,203
				36,149	36,24	36,752	36,736	36,123	36,489
				36,177	36,289	36,671	36,338	36,001	36,489
				36,391	36,429	36,617	36,307	35,933	36,62
				36,839	36,548	36,604	36,419	37,461	36,975
				36,584	36,129	36,523	36,68	37,449	37,243
				36,68	36,078	36,496	36,705	37,425	37,311
				36,722	36,204	36,496	36,87	37,412	37,325
				36,632	36,895	36,435	37,374	37,473	37,35
				36,88	36,916	36,435	36,957	37,443	37,3
				36,722	37,021	36,422	36,416	37,119	36,977
				36,536	36,77	36,361	36,191	36,795	36,566
				36,391	36,916	36,489	35,892	36,649	36,528
				36,294	36,944	36,294	35,867	36,594	36,46

				36,273	36,867	36,368	35,973	36,361	36,304
				36,136	36,546	36,489	36,023	36,276	36,31
				36,073	36,358	36,429	36,023	36,251	36,248
				35,991	36,225	36,53	36,198	36,6	36,279
				35,653	36,155	36,462	36,079	36,422	36,366
				35,529	36,085	36,334	36,042	36,062	36,123
				35,411	35,743	36,125	36,148	35,964	36,179
				35,294	35,631	36,139	36,104	36,092	36,217
				35,315	35,673	36,348	35,948	36,056	36,59
				35,287	35,638	36,523	35,574	35,909	37,145
				35,522	35,568	37,198	35,599	35,891	37,07
				35,598	35,421	37,292	35,911	35,842	37,319
				35,777	35,638	36,988	35,93	35,812	37,3
				35,818	35,533	36,786	36,011	36,093	37,331
				35,908	35,505	36,874	35,905	37,113	37,363
				36,218	35,547	36,739	36,104	38,011	37,331
				36,487	35,862	36,591	36,615	38,017	37,194
				36,894	35,855	36,469	36,933	37,895	37,095
				37,218	36,141	36,449	37,126	37,755	37,213
				37,342	36,84	36,948	37,474	37,431	36,921
				37,46	36,989	37,548	37,182	37,413	37,282
				37,474	37,275	37,926	37,388	37,352	37,462
				37,46	37,45	38,061	37,239	36,9	37,338
				37,418	37,317	38,162	37,463	36,997	36,883
				37,267	37,164	37,798	37,382	37,517	36,441
				37,239	36,996	37,805	37,308	37,596	36,086
				37,218	36,877	37,764	37,451	37,614	36,111
				36,529	37,296	37,218	37,196	37,565	36,055
				36,846	36,556	37,137	37,108	37,761	36,553
				36,515	36,612	37,123	37,152	37,229	36,964
				36,198	36,821	36,921	37,115	37,046	37,319
				36,018	36,877	36,975	37,14	37,126	37,562
				35,956	36,71	36,665	37,034	37,419	37,232
				35,908	36,423	36,577	36,778	37,016	37,275
				35,77	36,109	36,597	36,467	36,667	37,294
				35,556	35,955	36,665	36,305	36,582	37,151
				35,481	35,864	36,638	36,056	36,355	36,896
				35,398	35,836	36,631	35,906	36,239	36,628
				35,301	35,85	36,618	35,881	36,153	36,522
				35,232	35,746	36,557	35,676	35,952	36,316
				35,17	35,801	36,523	35,626	35,805	36,186
				35,232	35,878	36,476	35,632	35,554	36,186
				35,618	35,934	36,483	35,62	35,597	35,887
				35,708	35,99	36,341	35,925	35,487	35,562
				35,832	36,018	36,435	35,981	35,243	35,351

				35,873	36,018	36,489	35,838	35,481	35,394
				35,804	35,976	36,402	35,962	35,683	35,151
				35,694	35,913	36,408	35,969	35,787	34,864
				35,777	36,057	36,044	35,8	35,799	35,326
				35,908	36,05	36,004	35,37	35,628	35,623
				35,908	35,812	36,051	35,451	35,726	35,704
				36,039	35,742	35,909	35,539	35,585	35,785
				35,97	35,77	35,849	35,458	35,762	35,704
				35,949	35,889	35,889	35,501	35,872	35,729
				35,887	35,645	36,233	35,551	35,982	35,711
				35,825	35,526	36,341	35,557	36,612	35,729
				35,763	35,687	36,321	35,632	37,107	35,935
				35,666	35,68	36,314	36,168	37,217	36,165
				35,694	35,533	36,132	36,698	37,394	36,67
				35,804	35,596	36,213	36,741	37,394	37,268
				35,659	35,645	36,112	36,716	36,991	37,249
				35,625	35,61	36,118	36,411	37,473	37,118
				35,666	35,652	36,145	35,938	37,29	36,732
				35,528	35,631	36,294	35,589	37,437	36,44
				35,418	35,512	36,159	35,919	37,009	36,215
				35,314	35,519	36,139	35,919	36,538	36,053
				35,466	35,631	36,152	35,894	36,165	35,873
				35,453	35,61	35,99	36,255	35,915	36,016
				35,522	35,77	36,044	36,093	35,853	36,215
				35,425	35,749	36,523	36,006	35,823	36,271
				35,535	36,022	36,328	35,9	35,67	36,109
				35,342	36,05	36,489	35,8	35,664	36,035
				35,46	36,308	36,328	35,601	35,603	35,935
				35,37	36,085	36,469	35,532	35,578	35,873
				35,522	35,756	37,13	35,514	35,664	35,76
				35,556	35,882	37,386	35,545	35,621	35,648
				35,411	36,058	37,548	35,464	35,835	35,605
				35,542	36,184	37,359	35,627	35,902	35,636
				35,529	36,086	37,076	35,92	35,847	35,711
				35,804	36,107	36,759	35,939	35,878	35,792
				36,122	35,862	36,847	35,684	36,006	35,929
				35,777	35,897	36,584	35,528	36,031	35,785
				35,68	35,869	36,361	35,222	36,116	35,748
				35,929	35,799	36,334	35,247	35,994	35,736
				36,094	36,051	36,193	35,615	35,847	35,723
				36,066	35,911	36,274	35,677	35,438	35,748
				36,101	35,653	36,301	35,64	35,652	35,785
				35,839	36,295	36,314	35,596	35,762	35,842
				35,915	36,498	36,226	35,603	35,78	35,873
				36,528	36,212	36,388	35,709	36,214	35,935

				37,184	36,323	36,462	35,54	36,263	36,103
				37,17	36,191	36,624	35,627	36,33	36,458
				37,218	36,254	37,292	35,808	36,618	36,888
				36,866	36,142	37,71	35,777	36,606	36,994
				36,84	36,267	37,852	35,615	36,581	37,293
				36,543	36,442	37,838	35,571	36,801	36,888
				36,164	36,449	37,96	35,659	37,559	36,502
				36,136	36,582	37,488	35,958	37,84	36,521
				35,805	36,603	37,117	36,251	37,956	36,259
				35,84	36,777	36,901	35,883	37,901	36,028
				35,84	37,113	36,726	36,413	37,651	36,047
				36,254	36,77	36,415	36,593	37,113	35,96
				36,481	37,008	36,328	37,197	36,71	36,109
				36,916	37,252	36,233	36,755	36,551	36,072
				37,129	37,205	36,638	36,774	36,422	36,165
				37,323	37,128	37,171	36,363	36,545	36,844
				37,198	37,268	37,596	36,238	36,447	36,77
				36,992	37,436	37,366	36,456	36,691	37,181
				37,026	37,422	37,447	36,923	36,972	36,95
				36,647	37,45	37,542	36,674	37,052	36,9
				36,536	36,975	37,784	35,958	37,528	37,224
				36,481	36,528	37,886	36,089	37,62	37,324
				36,13	36,695	37,319	36,849	37,321	36,819
				36,054	36,87	37,056	36,637	37,064	36,869
				36,343	36,646	36,935	36,35	36,936	36,876
				36,219	36,737	37,063	36,575	36,777	36,595
				36,143	36,583	37,043	36,276	36,594	36,545
				36,212	36,632	36,746	36,282	36,306	36,577
				36,778	36,681	37,022	36,082	36,092	36,552
				37,157	37,03	37,805	35,977	36,215	36,477
				37,447	37,401	38,041	35,796	36,746	36,091
				37,447	37,128	38,263	35,983	37,644	36,882
				37,427	37,198	38,351	36,506	37,986	37,305
				37,482	37,359	38,344	36,736	37,98	37,486
				37,495	37,324	38,095	36,743	37,785	37,38
				37,371	37,519	38,115	36,805	37,925	37,511
				36,799	37,624	38,068	37,11	37,858	37,741
				36,482	37,589	37,859	37,116	37,895	37,784
				36,164	37,261	38,041	36,836	37,815	37,511
				36,137	37,01	37,798	36,929	37,87	38,027
				36,371	37,359	37,663	36,68	37,883	37,952
				36,716	37,212	37,704	36,68	37,51	37,399
				37,033	37,128	37,582	36,923	36,887	37,349
				36,523	37,009	37,36	36,794	37,26	37,201
				36,496	37,31	37,15	36,738	37,223	36,771

				36,806	37,17	36,887	36,738	37,034	37,238
				36,895	37,282	37,245	36,819	37,076	37,014
				37,089	37,491	37,825	37,062	37,009	37,226
				37,882	38,225	38,594	37,062	37,479	37,375
				38,013	38,343	38,803	37,547	38,219	38,091
				37,999	38,364	38,783	38,325	38,628	38,539
				37,654	37,666	38,574	38,368	38,518	38,551
				37,633	37,407	38,965	38,231	38,341	38,34
				37,709	37,491	38,978	37,92	37,779	38,004
				37,62	37,344	38,931	37,093	37,583	37,394
				37,571	37,072	38,749	37,031	37,504	37,338
				37,551	37,261	38,776	37,124	37,614	37,412
				37,833	37,694	38,871	37,199	37,663	37,369
				37,958	37,442	38,904	37,622	37,828	37,568
				38,137	37,861	39,066	37,18	37,791	37,78
				38,172	38,113	39,093	37,336	38,036	37,842
				38,144	37,826	38,938	37,572	38,036	37,755
				38,42	37,603	38,999	37,336	39,165	38,178
				38,275	37,386	39,005	37,622	39,245	37,898
				38,275	37,303	38,796	37,267	39,202	38,004
				38,137	37,351	38,749	37,522	39,19	38,041
				38,309	37,135	38,567	37,168	39,092	37,997
				38,185	37,519	38,5	37,112	39,159	38,066
				38,275	37,4	38,709	37,485	39,403	38,084
				38,247	37,868	38,486	37,118	39,184	38,06
				38,206	37,323	38,439	37,672	38,622	37,829
				38,178	37,281	38,432	37,149	38,964	37,748
				38,406	37,449	38,466	37,224	39,196	37,375
				38,165	37,414	38,79	37,541	39,391	37,78
				38,351	37,246	38,817	37,497	39,269	38,159
				38,447	37,372	38,83	37,578	38,86	38,103
				38,365	37,128	38,79	37,361	38,903	37,898
				38,158	37,365	38,844	37,566	39,068	37,966
				38,275	37,009	38,823	37,566	38,994	37,916
				38,144	37,197	38,79	37,529	38,799	37,817
				38,027	37,484	38,864	37,224	38,604	37,879
				38,206	37,26	38,877	37,193	38,61	37,692
				38,123	37,435	38,796	37,373	38,5	38,047
				37,896	37,651	38,776	37,161	38,555	37,761
				38,144	37,505	38,634	37,435	38,176	37,443
				38,109	37,386	38,25	37,249	38,25	37,45
				37,799	37,1	38,945	37,329	37,803	37,923
				37,909	37,442	38,513	36,869	37,479	37,842
				37,73	37,519	38,864	36,956	38,133	37,786
				37,806	37,162	38,324	37,298	37,718	37,68

				37,688	36,981	38,365	37,143	37,803	37,269
				37,102	37,128	38,54	37,093	37,699	37,63
				37,075	37,421	38,23	37,292	37,938	37,885
				37,026	37,197	38,25	37,292	37,815	37,773
				37,219	37,218	38,351	37,454	37,467	37,767
				37,337	37,686	38,338	37,242	37,15	37,705
				37,716	37,617	38,715	36,937	37,034	37,363
				37,909	37,344	38,486	36,937	37,253	37,493
				37,661	37,183	38,486	37,149	37,797	37,668
				37,888	37,232	38,533	37,068	38,219	37,761
				37,916	37,434	38,25	37,093	37,278	37,356
				37,847	37,127	38,446	37,155	37,461	37,549
				37,964	37,267	38,473	37,161	37,974	37,643
				37,978	36,841	38,365	37,168	37,895	37,668
				37,778	37,148	38,58	37,149	37,663	37,182
				37,882	37,162	38,742	37,255	37,999	37,375
				38,144	37,19	38,52	37,155	38,011	37,475
				37,937	37,078	38,392	37,118	38,17	37,363
				38,054	37,253	38,358	37,149	37,919	37,387
				38,019	37,127	38,58	37,398	37,473	37,711
				37,992	37,183	38,331	37,249	37,357	37,219
				37,875	37,225	38,216	36,601	37,339	37,145
				37,799	37,057	38,102	36,719	37,034	36,952
				37,84	37,497	38,142	36,495	37,076	37,406
				37,957	36,673	38,25	36,701	37,486	37,307
				37,861	37,204	38,048	36,906	37,663	37,456
				37,682	36,973	38,142	36,788	38,237	37,736
				37,744	36,939	38,088	37,255	38,249	37,524
				37,482	37,162	38,365	37,497	37,858	37,313
				37,764	37,05	38,371	37,796	37,492	37,226
				37,744	37,351	38,526	37,168	37,687	37,344
				37,833	37,476	38,371	37,18	38,048	37,387
				37,813	37,141	38,459	37,062	38,579	37,481
				37,482	37,414	38,439	36,738	38,157	37,325
				37,895	37,19	38,493	37,217	37,828	37,331
				38,102	37,057	38,405	37,211	37,724	37,226
				37,964	37,24	38,297	36,993	37,412	37,157
				37,689	37,275	38,054	36,975	37,467	37,313
				37,613	37,568	37,393	37,124	37,828	37,661
				37,509	37,38	37,022	37,118	37,388	37,375
				37,606	37,212	37,002	37,012	37,84	37,437
				37,771	36,989	36,894	37,031	37,705	37,219
				37,889	37,051	37,076	37,161	37,589	36,983
				37,675	37,198	37,535	36,775	37,822	37,107
				37,413	36,982	37,616	36,744	37,589	37,269

				37,737	36,968	37,825	37	38,451	37,319
				37,978	37,01	37,791	37,087	38,469	37,338
				37,778	37,254	38,324	36,894	38,151	37,493
				37,647	37,003	38,27	37,093	37,846	37,307
				37,599	37,415	38,203	36,937	38,28	36,945
				37,502	36,618	38,189	37,087	37,809	37,201
				37,247	36,164	38,182	37,136	38,139	36,914
				36,833	35,787	38,101	37,043	37,706	36,964
				36,537	35,417	37,65	37,124	37,627	37,12
				36,108	35,647	38,142	37,124	37,993	36,939
				35,922	35,508	37,636	37,149	37,84	36,466
				35,832	35,612	38,115	36,944	37,773	36,242
				35,75	36,171	38,054	36,819	37,401	36,179
				35,577	35,794	37,859	36,825	37,816	36,223
				35,591	35,864	37,784	36,769	37,553	36,21
				35,66	35,808	37,967	36,987	37,871	35,986
				35,646	35,696	37,717	37,193	37,572	36,13
				35,832	35,626	37,44	36,377	37,071	36,478
				35,722	35,605	37,798	36,308	36,716	36,341
				35,977	35,742	37,548	36,377	36,563	36,273
				35,826	35,923	37,312	36,301	36,374	36,109
				35,791	35,979	37,117	36,188	36,252	36,172
				35,777	36	37,029	35,908	36,081	36,29
				35,763	36,049	36,881	35,939	36,264	36,178
				35,77	35,965	37,016	35,659	36,142	36,296
				35,577	35,728	36,786	35,889	36,233	36,371
				35,35	35,644	36,746	35,921	36,276	36,315
				35,315	35,672	36,739	35,796	36,368	36,327
				35,564	35,714	36,456	35,821	36,368	36,402
				35,895	35,777	36,665	35,715	36,386	36,234
				36,15	36,335	37,056	35,958	35,977	36,035
				36,322	36,852	37,042	35,902	36,527	36,851
				36,688	37,076	37,11	36,338	37,095	36,913
				36,908	37,327	37,461	36,294	37,565	37,293
				37,129	37,684	37,568	36,743	37,559	37,081
				36,881	36,894	37,582	36,874	37,7	37,585
				36,612	36,845	37,697	37,141	37,572	37,504
				36,219	36,489	37,541	37,067	37,669	37,299
				35,984	36,293	37,366	36,948	37,431	37,411
				35,77	36,189	37,346	36,786	36,955	37,498
				35,826	36	37,103	36,818	36,588	37,293
				35,819	35,853	36,995	36,761	36,191	36,994
				35,812	35,777	36,833	36,531	36,233	36,695
				35,75	35,693	36,779	36,407	36,13	36,427
				35,598	35,804	36,557	36,307	36,032	36,016

				35,474	35,756	36,563	35,964	36,019	35,835
				35,377	35,846	36,55	35,964	35,952	35,686
				35,474	35,644	36,577	36,294	36,026	35,692
				35,343	35,435	36,671	36,262	36,026	35,399
				35,446	35,47	36,584	35,994	35,848	35,318
				35,405	35,386	36,732	35,825	35,806	35,486
				35,626	35,379	36,806	35,85	35,903	35,655
				35,777	35,26	36,806	35,819	35,812	36,184
				35,667	35,511	36,887	35,657	35,824	36,826
				35,626	35,721	37,339	35,963	36,074	36,882
				36,115	36,119	37,582	36,343	36,362	36,732
				36,66	36,874	37,784	36,517	36,912	36,944
				37,336	37,153	38,047	36,654	37,443	37,056
				37,501	37,502	38,047	36,785	37,669	37,318
				37,453	37,537	38,243	36,654	37,804	37,336
				37,35	37,433	37,892	37,115	38,634	37,318
				37,357	37,426	37,919	36,772	38,561	37,249
				37,253	37,118	37,852	36,617	37,926	37,286
				37,094	36,818	38,034	36,561	37,406	36,944
				36,667	36,65	37,879	36,449	36,771	36,651
				36,308	36,692	37,697	36,162	36,459	36,228
				35,922	36,762	37,319	36,361	36,386	35,804
				35,694	36,601	37,252	36,305	36,3	35,923
				35,694	36,448	37,083	36,237	36,312	35,567
				35,825	36,245	37,043	36,187	36,129	35,499
				35,853	36,042	36,908	35,975	36,306	35,524
				35,805	36,091	36,921	35,589	36,043	35,636
				35,75	35,903	36,894	35,738	35,891	35,692
				35,922	36,098	36,854	35,258	35,86	35,679
				35,908	36,056	36,732	35,196	36,153	35,692
				35,639	35,435	36,692	35,9	36,691	35,586
				35,446	35,449	36,645	35,489	37,473	35,923
				35,563	35,393	36,611	35,489	37,944	36,203
				35,494	35,504	36,537	35,969	37,901	36,925
				35,522	35,232	36,55	36,38	38,029	37,305
				35,563	35,847	36,692	36,318	37,846	37,324
				35,439	36,231	36,543	36,86	38,029	37,237
				35,322	36,755	36,577	36,791	37,925	37,573
				35,15	36,65	36,766	36,779	37,877	37,336
				35,343	36,161	37,373	36,685	37,443	37,156
				35,846	35,931	37,366	36,679	37,644	37,062
				36,136	35,588	37,272	36,716	37,382	36,882
				36,743	36,042	37,73	36,536	37,669	37,255
				37,053	36,804	37,906	36,828	37,773	36,788
				37,336	37,062	38,034	36,779	37,528	36,496

				37,37	37,195	37,933	36,455	37,669	36,222
				37,301	37,139	37,791	36,361	37,266	36,134
				37,274	37,034	37,757	36,343	36,979	36,172
				37,143	37,153	37,676	36,287	36,642	36,159
				37,157	37,111	37,548	36,274	36,532	36,203
				37,281	37,006	37,589	36,287	36,349	36,085
				37,302	37,048	37,609	36,1	36,245	35,873
				37,364	37,104	37,555	36,125	36,001	36,184
				37,268	36,86	37,272	36,037	35,842	36,053
				37,081	36,706	37,009	36,299	35,781	35,767
				36,55	36,622	36,874	36,473	35,524	35,823
				36,309	36,42	36,732	36,486	35,273	35,704
				35,978	36,364	36,611	36,361	35,298	36,066
				35,833	36,191	36,476	36,38	35,243	35,785
				35,73	36,009	36,462	36,218	35,151	35,661
				35,647	35,806	36,53	36,044	35,224	35,817
				35,654	35,862	36,476	36,343	35,181	35,773
				35,647	35,667	36,327	36,006	35,151	35,717
				35,619	35,855	36,179	36,106	35,108	35,679
				35,537	35,869	36,044	35,944	35,634	35,717
				35,364	35,716	36,037	35,944	35,946	35,642
				35,33	35,792	36,085	35,938	36,673	35,679
				35,185	35,988	36,206	35,607	36,905	35,704
				35,088	36,002	35,977	35,489	37,302	35,742
				35,102	36,156	36,192	35,695	36,972	35,48
				35,109	35,674	35,956	35,632	36,63	36,06
				35,171	35,862	36,186	35,539	36,337	36,128
				35,143	35,89	36,186	35,545	36,08	36,097
				35,206	35,911	36,112	35,738	35,982	35,835
				35,24	35,772	35,889	35,713	35,854	35,817
				35,199	35,855	36,341	35,663	35,836	35,823
				35,142	35,82	36,327	35,657	35,719	35,972
				34,963	35,457	36,125	35,763	35,762	35,979
				34,963	35,716	36,024	35,514	35,781	35,736
				35,142	35,618	35,916	35,682	35,848	35,829
				35,28	35,702	35,727	35,501	35,793	36,489
				35,425	35,702	35,687	35,607	35,823	36,296
				35,363	35,555	35,693	35,526	35,781	36,184
				35,446	35,737	35,747	35,551	35,664	35,904
				35,542	35,792	35,849	35,931	35,768	35,823
				35,439	35,834	35,923	35,919	35,823	35,86
				35,466	35,799	35,801	35,688	35,964	35,698
				35,597	35,715	35,734	35,252	36,282	35,617
				35,604	36,232	36,024	35,589	36,062	36,246
				35,625	36,421	36,712	35,938	36,392	36,153

				35,549	36,588	36,88	36,056	37,26	36,795
				35,46	36,351	37,063	36,492	37,705	36,701
				35,46	36,665	37,474	36,417	37,736	36,446
				35,646	36,665	37,245	36,368	37,455	36,464
				35,66	36,623	36,941	35,994	37,687	36,489
				36,066	36,302	36,644	35,944	37,394	36,19
				36,184	36,162	36,462	35,72	37,07	36,028
				36,322	36,33	36,786	35,744	36,673	35,842
				36,666	36,561	37,002	35,794	36,312	35,81
				36,784	36,896	37,123	35,95	36,068	35,754
				36,915	37,022	37,542	35,894	35,842	35,729
				36,701	37,406	37,15	35,925	35,866	35,935
				36,88	37,301	37,069	35,857	35,854	35,947
				36,349	36,665	36,874	35,726	35,86	35,985
				35,887	36,798	36,887	35,564	35,927	35,817
				35,639	36,791	36,813	36,15	36,16	36,153
				35,37	36,498	36,705	36,143	36,117	36,564
				35,287	36,616	36,557	36,305	36,227	36,844
				35,218	36,54	36,206	36,473	35,988	36,988
				35,218	36,372	36,381	36,623	36,374	37,212
				34,991	35,918	36,294	36,922	36,887	37,33
				35,177	36,092	36,53	36,455	37,412	37,442
				35,177	36,302	36,8	36,629	37,846	37,38
				35,68	36,889	37,137	36,343	37,901	37,212
				36,611	36,939	37,912	36,287	37,889	37,106
				36,922	37,351	38,203	36,35	37,925	36,945
				37,274	37,428	38,25	36,755	38,255	37,3
				37,487	37,637	38,364	36,749	37,858	37,288
				37,66	37,679	38,668	36,979	37,974	37,294
				37,453	37,637	38,358	37,116	37,516	37,375
				36,653	37,106	37,4	37,378	38,322	37,064
				35,439	36,24	36,537	37,558	38,707	37,761
				34,86	35,43	35,869	38,081	39,232	38,004
				33,818	34,389	35,316	38,193	39,079	37,966
				32,653	33,138	34,54	37,962	38,884	37,717
				31,798	32,167	33,393	37,552	39,024	37,674
				31,177	31,657	33,09	37,459	38,695	37,755
				30,632	31,21	32,773	37,62	38,518	37,91
				30,101	30,84	32,463	37,446	38,182	37,661
				29,639	30,533	32,186	37,595	38,2	37,356
				29,26	30,232	31,93	37,527	38,023	37,257
				28,929	29,988	31,694	37,16	38,689	37,089
				28,674	29,827	31,512	37,116	38,084	36,989
				28,488	29,729	31,357	37,614	38,157	37,163
				28,343	29,715	31,141	37,757	37,803	36,97

				28,343	29,953	31,053	37,558	37,278	36,827
				28,467	30,1	30,972	37,577	37,088	36,927
				28,633	30,176	30,932	37,627	37,04	37,057
				28,991	30,267	30,945	37,421	37,095	37,213
				29,288	30,239	30,864	37,272	37,156	37,039
				29,86	30,609	30,992	37,073	37,131	36,97
				30,488	31,245	31,154	36,786	37,174	36,958
				31,178	31,98	31,357	37,191	37,241	36,877
				31,709	32,853	31,491	37,371	37,956	36,894
				32,157	33,468	32,159	37,104	38,237	37,324
				32,55	34,187	32,652	37,085	38,371	37,685
				32,998	35,2	33,306	37,558	38,505	37,859
				33,564	35,536	33,839	37,44	37,864	38,077
				34,364	35,899	34,358	37,228	37,449	37,392
				34,977	36,025	34,83	37,147	37,125	37,087
				35,212	35,871	35,08	37,147	37,058	36,701
				35,288	35,654	35,383	37,104	36,814	36,651
				35,219	35,99	35,626	36,998	36,716	37,23
				35,115	36,437	36,166	36,774	36,807	36,981
				35,398	36,283	36,132	36,606	37,003	37,33
				35,791	36,569	35,984	36,755	37,449	37,535
				35,798	36,541	35,943	36,537	37,901	37,311
				35,571	37,296	36,004	36,811	38,292	37,342
				35,419	37,422	36,274	36,93	37,767	37,349
				35,784	36,73	36,206	36,967	37,986	37,131
				35,96	36,919	36,53	36,724	37,406	37,187
				36,209	36,912	36,975	36,824	37,602	37,05
				36,147	37,422	37,022	36,824	38,481	37,243
				36,126	37,135	37,204	37,328	37,822	37,591
				36,195	36,94	37,353	37,129	37,449	37,716
				36,395	36,772	37,346	36,998	37,418	37,604
				36,485	37,24	37,629	36,849	38,102	37,56
				36,347	37,785	38,034	37,166	38,151	37,747
				36,319	37,373	38,027	37,029	38,542	37,542
				36,25	37,492	37,973	36,73	37,968	37,423
				36,34	37,535	37,832	37,334	37,687	37,361
				36,381	37,22	37,73	37,322	37,913	37,655
				36,595	37,095	37,919	36,861	38,072	37,68
				36,878	37,891	38,344	36,78	37,962	37,593
				37,002	38,066	38,176	36,799	38,261	37,593
				37,009	37,884	38,095	36,979	38,707	37,786
				36,857	37,604	38,169	37,235	37,962	38,01
				36,857	37,458	38,418	37,278	37,644	37,742
				36,816	37,556	38,344	37,166	38,048	37,979
				37,078	37,716	38,594	37,465	38,451	37,823

				37,34	37,598	38,763	37,365	38,17	37,817
				37,533	38,1	38,79	37,521	37,907	38,047
				37,547	38,017	38,473	37,465	38,194	38,053
				37,519	37,849	38,844	37,471	39,006	38,053
				37,519	37,884	38,641	37,633	38,609	38,159
				37,547	37,653	38,527	37,571	37,993	38,215
				37,25	37,884	38,547	37,664	38,347	38,116
				37,236	37,821	38,722	37,689	38,475	38,377
				37,292	37,563	38,594	37,801	37,914	38,445
				37,223	37,709	38,715	36,973	37,846	38,364
				37,492	37,528	38,432	37,446	38,359	38,004
				37,885	37,416	38,682	37,06	38,219	37,705
				37,961	37,863	38,837	37,39	37,822	37,562
				38,065	38,017	38,999	37,546	38,555	37,811
				37,678	37,723	39,154	37,123	38,207	37,842
				37,375	37,8	39,356	37,564	38,591	38,053
				37,368	37,646	38,938	37,297	38,482	38,284
				37,596	37,765	39,059	37,539	37,846	38,402
				37,672	38,094	38,904	37,577	38,567	38,564
				37,403	38,136	38,749	37,595	38,512	38,21
				37,209	37,969	39,046	37,515	38,133	38,434
				37,092	37,668	38,648	37,527	38,17	38,235
				37,072	37,396	38,783	37,241	38,329	37,837
				37,065	37,41	38,54	36,818	38,64	38,167
				37,078	37,179	38,871	37,9	38,274	38,459
				37,251	36,914	38,999	37,739	37,639	38,521
				37,616	37,354	39,026	37,502	37,828	38,241
				37,796	37,487	39,585	37,409	37,889	38,129
				37,396	37,396	39,7	37,471	38,097	38,409
				37,099	37,438	39,107	37,819	38,048	38,192
				36,844	37,032	38,837	37,714	37,749	38,21
				36,809	36,893	38,931	37,552	37,431	38,154
				36,899	37,333	39,019	37,658	37,865	37,737
				37,044	37,815	39,356	37,465	38,408	37,793
				36,816	37,556	39,093	37,564	37,975	38,117
				37,023	37,494	38,661	37,054	37,816	38,148
				37,099	37,452	39,262	37,123	37,633	38,042
				37,506	36,963	39,073	37,154	37,846	38,08
				37,713	37,046	39,268	37,253	38,573	37,719
				37,954	37,417	39,262	37,515	38,237	37,669
				38,023	37,514	39,039	37,421	37,443	37,787
				38,058	37,563	38,978	37,701	37,926	38,098
				37,644	38,052	38,614	37,776	37,889	37,874
				37,271	37,542	38,149	37,515	38,347	37,482
				37,058	37,431	38,331	37,154	37,401	37,75

				36,933	37,591	38,223	37,869	37,303	37,644
				36,83	37,393	38,358	37,614	37,327	37,127
				36,665	37,135	38,466	37,384	37,804	37,014
				36,699	37,219	38,634	37,116	37,859	37,057
				36,527	37,386	38,54	37,123	37,993	36,889
				36,54	37,24	39,221	37,832	38,121	37,263
				36,775	36,876	38,871	37,633	37,853	37,437
				37,382	37,365	38,783	37,62	37,743	37,4
				37,561	37,365	38,884	37,564	38,091	37,151
				37,961	37,659	39,012	37,359	38,366	37,381
				37,927	37,715	38,776	37,035	38,14	37,78
				37,175	37,659	38,371	36,743	37,816	37,717
				37,402	37,624	38,041	36,363	37,859	37,375
				36,74	37,128	38,007	36,344	37,584	37,319
				36,789	37,31	37,967	36,095	37,083	37,381
				36,554	37,505	37,953	35,889	37,529	37,325
				36,582	37,407	37,731	35,441	37,333	36,97
				36,651	37,568	37,717	35,79	37,113	37,3
				37,078	37,303	37,292	36,095	37,412	36,497
				36,934	37,107	37,717	36,332	37,443	36,846
				37,416	36,974	37,704	37,247	37,461	37,02
				37,637	37,058	38,189	37,546	37,632	36,447
				37,713	37,407	38,304	37,539	37,718	36,877
				37,899	37,296	38,891	37,291	37,852	36,752
				38,182	37,645	39,039	37,714	38,048	36,485
				37,885	37,435	38,83	37,763	37,779	36,509
				37,265	37,973	38,628	37,639	37,644	36,429
				37,003	37,421	37,946	37,595	37,431	36,08
				36,685	37,526	38,156	37,595	37,425	36,086
				36,582	37,729	38,203	37,079	37,321	35,793
				36,589	37,247	38,378	36,73	37,229	35,748
				36,306	36,933	37,92	36,656	36,82	35,686
				36,354	37,038	37,812	37,409	36,697	35,58
				36,41	36,758	37,596	37,216	36,655	35,642
				36,43	36,423	37,717	36,624	36,526	35,717
				36,23	36,311	37,387	36,718	36,734	35,742
				35,851	36,102	37,056	36,101	36,771	35,91
				35,603	36,444	37,204	35,771	36,704	36,022
				35,417	36,682	37,036	35,571	36,661	36,116
				35,817	36,542	36,82	35,615	36,398	36,085
				35,527	36,325	37,164	35,571	36,27	35,362
				35,658	36,444	37,258	35,547	36,117	35,218
				36,182	36,381	37,029	35,422	36,092	35,318
				36,168	36,423	36,928	35,584	36,202	35,486
				36,086	36,242	36,914	35,596	36,416	35,281

				35,858	36,332	36,941	35,596	37,467	35,062
				35,741	36,346	36,8	35,621	37,504	36,483
				35,733	36,339	36,732	35,534	37,803	36,876
				35,622	36,242	36,752	35,603	38,06	37,299
				35,781	36,689	37,305	35,578	37,583	37,286
				36,16	37,045	37,879	35,727	37,571	37,417
				36,167	37,213	38,31	35,684	37,596	37,318
				35,96	37,52	38,567	35,871	37,425	37,492
				36,257	37,771	38,56	35,927	37,699	37,006
				36,34	37,618	38,439	35,921	37,156	36,321
				36,271	37,164	37,859	36,207	36,783	35,823
				36,119	36,968	37,393	36,4	36,563	35,592
				36,112	36,784	37,049	36,363	36,331	35,337
				36,078	36,651	36,773	36,357	36,355	35,299
				35,912	36,533	36,705	36,294	36,288	35,206
				35,85	36,449	36,483	36,12	36,306	35,187
				35,809	36,47	36,368	36,058	36,251	35,362
				36,016	36,519	36,193	36,076	36,147	35,717
				36,374	36,232	36,348	36,026	36,031	35,817
				36,678	36,162	36,597	36,014	35,921	35,829
				36,76	36,281	37,076	35,896	35,885	35,76
				36,512	36,547	37,144	35,833	36,178	35,742
				36,133	37,294	37,501	35,646	36,618	35,748
				36,284	37,154	37,892	35,653	37,589	35,679
				36,96	37,036	38,385	35,59	38,304	35,455
				37,264	37,029	38,418	35,734	37,846	35,742
				37,76	37,35	38,648	35,945	37,101	35,785
				37,353	37,343	38,803	35,914	37,107	35,829
				36,726	37,601	38,628	36,332	37,504	36,415
				36,533	37,42	37,811	36,556	37,388	37,237
				36,306	37,266	37,38	37,179	36,936	37,647
				36,188	37,077	37,15	37,216	37,346	37,753
				36,05	37,147	36,982	36,861	37,193	37,492
				35,94	36,609	37,137	36,892	37,107	37,878
				35,83	36,267	37,137	37,017	37,449	37,716
				35,602	36,016	37,09	36,799	37,358	37,342
				35,471	36,023	36,901	37,147	37,058	37,598
				35,361	35,967	36,82	37,16	36,79	37,38
				35,402	35,841	36,678	37,023	36,588	37,262
				35,223	35,953	36,935	36,836	36,295	36,963
				35,244	35,972	36,928	36,712	36,154	36,72
				35,237	35,993	36,955	36,656	36,032	36,371
				35,112	35,818	36,948	36,357	35,903	36,035
				35,244	35,769	36,773	35,927	35,763	35,829
				35,216	35,804	36,86	35,478	35,793	35,798

				35,306	35,755	36,8	35,628	35,738	36,022
				35,623	35,825	36,719	35,584	35,683	36,078
				35,519	35,909	36,685	35,677	35,683	35,991
				35,74	35,972	36,563	35,883	35,781	35,742
				36,243	36,042	36,786	35,79	35,708	35,63
				36,864	36,286	37,973	35,896	35,824	35,549
				37,237	36,726	38,587	35,889	35,867	35,461
				37,733	37,027	38,749	35,808	35,903	35,642
				37,919	37,439	38,951	35,621	35,885	35,723
				37,919	37,886	38,931	35,827	35,958	35,711
				37,995	38,109	38,796	35,571	36,6	35,754
				37,85	38,026	39,18	35,528	36,911	36,384
				37,588	37,774	38,776	35,902	37,382	36,925
				37,182	37,592	37,987	36,475	37,528	37,23
				36,851	37,432	37,434	37,091	37,449	37,411
				36,417	37,229	37,076	36,917	37,522	37,56
				36,113	36,915	36,887	36,768	37,437	37,498
				35,886	36,747	36,705	36,836	37,131	37,448
				35,803	36,586	36,53	36,375	37,021	36,907
				35,672	36,559	36,55	36,855	36,966	36,813
				35,499	36,538	36,381	36,537	36,606	36,147
				35,258	36,475	36,341	36,431	36,331	35,848
				35,21	36,328	36,388	36,469	36,208	35,592
				35,286	36,245	36,334	36,961	36,001	35,686
				35,348	36,14	36,361	36,22	36,092	35,773
				35,161	36,168	36,442	35,621	36,068	35,742
				35,265	36,126	36,395	35,796	35,897	35,704
				35,217	35,938	36,253	35,939	35,854	35,76
				35,41	35,966	36,273	35,609	35,836	35,505
				35,534	35,91	36,159	35,584	35,842	35,362
				35,679	35,868	36,3	35,628	35,811	35,387
				35,679	36,035	36,226	35,653	35,805	35,424
				35,375	36,035	36,732	35,628	35,86	35,499
				35,106	36,161	37,407	35,422	35,756	35,399
				35,306	36,608	37,838	35,359	35,75	35,237
				35,472	36,762	38,277	35,285	35,64	35,025
				35,437	36,685	38,277	35,403	35,86	35,274
				35,472	37,02	38,074	35,26	36,178	35,306
				35,354	37,419	37,737	35,204	36,251	35,037
				35,189	37,614	37,393	35,172	36,856	35,773
				35,272	37,649	36,86	35,222	37,143	36,116
				35,353	37,922	37,002	35,135	37,216	36,346
				35,422	37,873	36,914	35,366	37,088	36,689
				35,65	37,342	36,86	35,565	37,167	36,944
				35,781	37,034	36,833	35,921	37,112	37,037

				35,629	36,587	37,056	35,952	37,387	37,025
				35,636	36,427	36,874	36,332	36,856	37,218
				35,664	36,217	36,651	36,481	36,611	36,938
				35,822	36,119	36,307	36,861	36,275	36,876
				35,609	35,959	36,408	36,631	36,074	36,876
				35,512	35,833	36,53	36,344	35,97	36,907
				35,34	35,699	36,476	36,226	36,116	36,844
				35,285	35,775	36,233	36,07	36,049	36,566
				35,319	35,908	36,267	35,958	36,098	36,341
				35,174	35,999	36,402	36,114	35,89	36,092
				35,643	36,062	36,402	36,132	35,578	35,862
				35,636	36,013	37,015	36,064	35,615	35,656
				35,485	35,915	37,042	35,889	35,853	35,544
				35,388	35,943	36,773	35,653	36,086	35,575
				35,333	36,153	36,779	35,802	35,988	35,419
				35,167	36,229	36,692	35,964	35,896	35,4
				35,057	36,118	36,631	35,715	35,603	35,6
				35,174	35,908	36,86	35,596	35,603	35,712
				35,271	36,006	37,488	35,584	35,872	35,619
				35,574	36,264	38,101	35,584		35,693

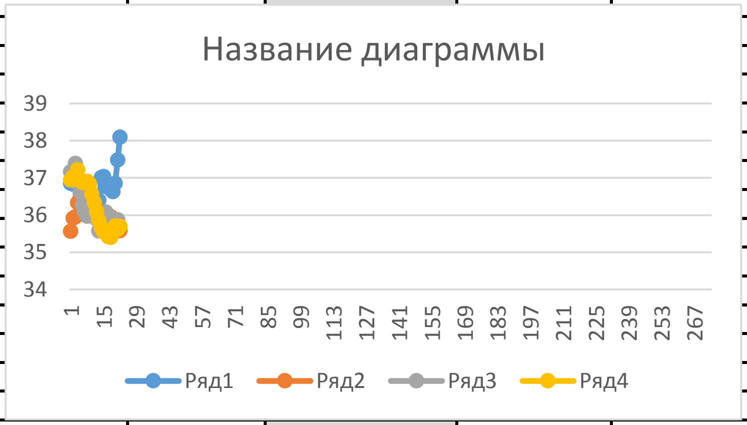
Название диаграммы
39
38
37
36
35
34
1
15
29
43
57
71
85
99
113
127
141
155
169
183
197
211
225
239
253
267
Ряд1
Ряд2
Ряд3
Ряд4

